# Development and validation of the “Adjustment Disorder Scale for Medically Ill Patients - ETAM”

**DOI:** 10.3389/fpsyt.2025.1482888

**Published:** 2025-01-30

**Authors:** Juan Pablo Zapata-Ospina, Natalia Rodríguez, Ayda Margarita Rodríguez, Jenny García-Valencia, Mercedes Jiménez-Benítez, Nicolás Martínez-Ramos, Diana Restrepo Bernal, Ana Lucía Gallego, Carolina Gómez, Luis Fernando Tabares, Carlos Cardeño-Castro, Daniel Camilo Aguirre-Acevedo

**Affiliations:** ^1^ Institute of Medical Research, Academic Group of Clinical Epidemiology (GRAEPIC), Faculty of Medicine, Universidad de Antioquia, Medellín, Colombia; ^2^ Department of Psychiatry, Faculty of Medicine, University of Antioquia, Medellín, Colombia; ^3^ Department of Psychology, Faculty of Social and Human Sciences, University of Antioquia, Medellín, Colombia; ^4^ Centro Javeriano de Oncología, Hospital Universitario San Ignacio, Bogotá, Colombia; ^5^ Faculty of Medicine, Universidad CES, Medellín, Colombia; ^6^ Department of Psychology, Universidad del Norte, Barranquilla, Colombia; ^7^ Hospital Alma Máter de Antioquia, Medellín, Colombia; ^8^ Department of Psychiatry, Hospital Universitario San Vicente Fundación, Medellín, Colombia

**Keywords:** adjustment disorders, emotional adjustment, validation studies, psychometrics, patient reported outcome measures, diagnosis

## Abstract

**Background:**

Adjustment disorder (AD) is common among medically ill patients, yet current evaluation methods do not address the specific characteristics in this population. This study aimed to develop a measurement scale for AD in medically ill patients in Colombia and to find evidence of its validity and reliability.

**Methods:**

This was a scale development and validation study. In the first qualitative phase, items were developed. In the second phase, the content validity of each item was evaluated by patients and clinicians. In the third phase, structural validity, internal consistency, test-retest reliability, criterion validity, and convergent construct validity were assessed. Items were analyzed using a generalized partial credit model within an item response theory framework.

**Results:**

The Adjustment Disorder Scale for Medically Ill Patients (ETAM, for its acronym in Spanish) was developed, comprising 20 items that address the free description of stressful situations in the last 15 days and mental symptoms attributed to them. Evidence of content validity was found. The scale was administered to 512 medically ill patients, revealing a three-dimensional structure: 1) “AD Symptoms”, 2) “Impact on Self-Care”, and 3) “Impact on Desire to Live”. Internal consistency was adequate, with McDonald’s omega of 0.95 and Cronbach’s alpha between 0.82 and 0.92 for its dimensions. ETAM had high test-retest reliability (intraclass correlation coefficient of 0.98). Criterion validity evidence was obtained with an independent psychiatrist’s diagnosis, with an AUROC of 0.99, and convergent validity was consistent with hypotheses of correlation with other instruments with similar constructs. Discrimination and difficulty parameters were calculated for each item.

**Conclusion:**

The ETAM is a scale with evidence of validity and reliability that can be used for the diagnosis of AD in medically ill patients in Colombia.

## Background

Adjustment disorder (AD) is defined as a reaction by an individual to a perceived stressor that is more intense or prolonged than what would typically be expected, given the nature of the stressor and the cultural context in which the person lives. This reaction not only exceeds the boundaries of what is considered a normal response within their cultural norms but also significantly interferes with their ability to function in daily life (1, 2). It is self-limiting as disorder is closely linked to a specific stressor or life event, and as the situation changes -whether because the stressor ends, or the individual develops coping mechanisms to handle it- the symptoms diminish or disappear (3). And the diagnosis of AD requires that the symptoms do not meet the diagnostic criteria for other mental disorders, such as a major depressive episode (MDE) (4). AD is of particular clinical interest as the risk of suicide in individuals with this diagnosis is 12 times higher than in the general population (5), and between 5% and 36% of those who have committed suicide were found to have AD through psychological autopsy (6–10). Furthermore, it involves work absenteeism and medical disabilities (11), generating significant costs, especially because AD primarily affects individuals in economically productive ages (12, 13).

Medically ill patients frequently present with AD. In high-complexity general hospitals, the proportion of patients hospitalized for medical illnesses who are evaluated by liaison psychiatrists and diagnosed with AD varies between 10.6% and 18.5% (14). In some clinical settings, the prevalence is notably high, particularly among patients facing significant medical illnesses such as cancer (with a prevalence of 23.5% and 38.6%) (15, 16), pulmonary arterial hypertension (38.2%) (17), cardiac disease (33%) (18), and infertility (60.1%) (19). It has also been found that AD leads to greater consumption of healthcare resources in this population and is associated with the development of complications (20).

A medical illness can be considered a stressful event because from the onset of symptoms, through diagnosis and treatment, emotional reactions can arise that cause suffering and impair functionality. Such reactions are considered abnormal and may be part of a range of diagnostic possibilities, including AD (21). Depressive symptoms, for instance, have a high prevalence in medical illnesses, particularly those affecting the gastrointestinal, hematological, renal, neurological, and cardiovascular systems (22), making them one of the main reasons for consultation in liaison psychiatry services.

From a clinical standpoint, diagnosing adjustment disorder (AD) in medically ill patients can be challenging and may be presented differently. Besides being a reaction to the illness and its consequences, mental symptoms can also be part of the underlying disease or an adverse effect of treatment (23). Additionally, an accurate diagnosis could guide different treatment approaches: in AD, psychotherapy aimed at coping with the stressor would be prioritized, whereas in MDE, treatment might focus on antidepressants (24, 25), which carry a higher risk of drug interactions and adverse effects in this population (26).

There are two instruments for measuring AD. One is the International Adjustment Disorder Questionnaire (IADQ), which has not been validated in medically ill patients (27). The other is the Adjustment Disorder New Module (ADNM), based on DSM-5 and ICD-11 criteria, and validated in this population. However, this scale excludes behaviors specific to disease management, focusing only on repetitive thoughts, avoidance, and maladaptation to stressors, with the latter defined as sleep and concentration disturbances (28). Medically ill patients might show difficulties in adapting to a stressful situation through neglecting their medical treatment, losing interest in continuing recommended therapy, or neglecting self-care activities (16), rather than just sleep and concentration disturbances.

Considering these characteristics of the instruments and the current definitions of diagnostic manuals, there is a clear need to improve the definition of AD for medically ill patients and to develop an instrument for its measurement. The objective of this study was to develop and validate a measurement instrument for the evaluation and diagnosis of AD in medically ill patients.

## Methods

Scale development and validation study conducted in three phases (29) following the COSMIN taxonomy (30). The study was carried out in two high-complexity hospitals in Medellín, Colombia. It complies with the Declaration of Helsinki and Colombian research standards and was approved by the Ethics Committee of the Faculty of Medicine at the University of Antioquia (Approval Act 008, May 2020) and the participating institutions. Each patient included in the study participated voluntarily and signed informed consent.

### Phase 1: item development

A Scale Development Group (SDG) was formed to create a measurement instrument for the evaluation and diagnosis of AD in medically ill patients, whether hospitalized or treated on an outpatient basis. The SDG included a psychiatrist specializing in liaison psychiatry, two epidemiologist psychiatrists with experience in psychometrics, a psychologist with a Ph.D. in clinical psychology, and a psychologist specialized in psychometrics. To enrich the discussions, we also included a female patient with chronic kidney disease who has participated in psychology research as a patient representative. The SDG held regular meetings, and the methodology was based on individual responses to questions before each session, followed by group discussions to share and analyze individual responses and reach a consensus (31). For the theoretical definition of the construct and items, results from two qualitative studies were used (32). One phenomenological study described the differential clinical characteristics of AD (33) and another grounded theory study explored the diagnostic criteria used by psychiatrists and psychologists for AD (34). With this input, the operational definition of AD and its conceptual domains were developed (35).

Based on this operational definition, in-depth interviews were conducted with seven adult patients with medical illnesses hospitalized in the two participating hospitals. These interviews provided empirical input for the construction of items from the target population (36). An interview guide was used, delving into how to ask about the conceptual domains of AD and the process of adapting to a medical illness. A psychiatrist epidemiologist trained in qualitative research conducted the interviews, which were recorded, transcribed verbatim, and then analyzed using grounded theory techniques to identify potential items in the words and expressions used by the patients, ensuring content validity of the items from their inception. The item pool was evaluated by the SDG in an iterative process to determine relevance, domain comprehensiveness, and comprehensibility. This step resulted in the first version of the scale.

### Phase 2: scale development

The first version of the scale underwent a content validity evaluation process with patients (37). Cognitive interviews were conducted to determine the patient’s understanding of each item, difficulties in responding, and any deviation from the construct while reasoning or responding (38). Initially, seven Colombian adult medically ill patients, both hospitalized and from outpatient settings, were included. Each item was presented to them with the instruction to “think aloud” to make their reasoning explicit while responding (39). Verbal probes were also used to assess the comprehensibility and exhaustiveness of the items, the acceptability of the questions, and the absence of discrimination. Memos were taken to note observable behaviors during their responses (40).

The cognitive interviews were recorded, transcribed verbatim, and analyzed line by line independently by two psychiatrists, who later met to discuss the comprehensibility evidenced. As a result, it was necessary to modify the phrasing of some items, which then had to be re-evaluated by a new group of seven patients through an iterative process of cognitive interviews and subsequent item revisions or eliminations due to lack of comprehensibility or relevance. Five rounds of cognitive interviews, each with seven patients, were required.

After the content evaluation by patients, the preliminary version of the scale was subjected to expert clinical review. This included 12 psychiatrists and psychologists experienced in evaluating medically ill patients, who assessed the relevance of each item for measuring the construct of AD and the comprehensiveness of the version. Each item was rated on a scale from 1 to 4 (1 = Not relevant, 2 = Slightly relevant, 3 = Relevant, 4 = Very relevant), and then dichotomized as either not relevant (values 1 and 2) or relevant (values 3 and 4) (41). The content validity index at the item level (CVI-I) was calculated and deemed adequate if it was greater than 0.78 (42).

Additionally, the scale-level average content validity index (S-CVI-Ave) was calculated, with values greater than 0.80 considered adequate (43), and Fleiss’ Kappa (κ) was used as a measure of agreement. Only after this evaluation did the SDG approve the final version of the instrument. A pilot test was then conducted with 20 medically ill adult patients to monitor the completion process, determine the average completion time, and identify missing data. The data from participants in the pilot test were included in the psychometric property evaluation.

### Phase 3: evaluation of psychometric properties

#### Participants

The inclusion criteria were: Colombian adults with a confirmed medical diagnosis, from any educational background, who were receiving outpatient or inpatient care at one of the two participating centers (excluding Intensive Care Unit and Special Care Unit), and those who agreed to participate after the informed consent process. Exclusion criteria were patients with a diagnosis of delirium, dementia syndrome, active psychosis or mania, intellectual disability, or language impairments that prevented effective communication.

A sample size was calculated for the evaluation of each psychometric property. For structural validity, the goal was to recruit at least 500 subjects, which is the recommended sample size for conducting factor analyses (44). Internal consistency was also assessed within this subsample. For item response theory (IRT) analysis, 500 participants were included as recommended by Ayala (45). Test-retest reliability was evaluated in 41 subjects, based on the sample size estimation formula described by de Vet (46), assuming an expected intraclass correlation coefficient (ICC) of 0.70, with a precision level of 0.20, type I error of 0.05, and type II error of 0.20.), For criterion validity, it was estimated that 204 subjects were needed to achieve an area under the ROC curve (AUROC) of 0.70, with a confidence interval width of 0.20, an expected AD prevalence of 18.5% in general hospitals (47) and a 95% confidence level (48). For convergent construct validity, a subsample of 65 subjects was calculated to seek moderate correlations with similar instruments, according to the formula for a Spearman’s correlation coefficient >0.50 in the alternative hypothesis and <0.10 in the null hypothesis (49).

#### Procedures

The scale was administered by trained physicians and nursing assistants. The scale was self-administered by the patient or, in cases where manipulating paper and pencil was difficult due to the presence of catheters or medical reasons, assisted administration was performed. For test-retest reliability, the same evaluator reapplied the scale to a subsample of participants three to four days after the initial application. This time frame was considered appropriate given the high variability of the construct and to prevent recall of the questions. The Clinical Global Impression (CGI) scale (50), completed by both the patient and the initial evaluator, was used as an anchor to ensure that patients remained stable during the interim period for the analysis of this property, given that AD can be fluctuating.

For criterion validity, the reference standard was an independent evaluation by a liaison psychiatrist conducted at the same hour the instrument was administered. The psychiatrist independently assessed the patient using a symptom checklist derived from the operational definition of the SDG. It was considered necessary to use the theoretical model developed in order to be consistent with the change in the definition of AD by placing it in the same hierarchy, as opposed to other diagnostic criteria or structured interviews. For convergent construct validity, the calculated subsample was administered the Hospital Anxiety and Depression Scale (HADS) (51) and the 8-item Adjustment Disorder New Module (ADNM-8) (52). For discriminant construct validity, the scores of patients diagnosed with AD were compared to those without AD.

#### Instruments

##### Hospital Anxiety and Depression Scale (HADS)

This is a 14-item self-report scale with three response options, designed to describe depressive and anxious symptoms experienced over the past week (51). It is one of the most commonly used tools for detecting emotional distress in individuals with physical illnesses (53). In Colombia, it has been validated in oncology patients, showing a structure consistent with separate anxiety and depression factors, and demonstrating adequate reliability and validity as a screening tool for anxiety and depression. Internal consistency has been measured with Cronbach’s alpha and was adequate for the anxiety (α=0.80 to 0.86) and depression (α=0.80 to 0.87) subscales (50).

##### New Adjustment Disorder Module (ADNM)

This is a self-report scale that originally includes 29 items (54). It begins with a list of stressful events potentially triggering adjustment disorder, where respondents select those experienced in the past year to reference for the rest of the scale. The 8-item version (ADNM-8) assesses adjustment disorder symptoms over the past two weeks, categorized into two dimensions: preoccupation (cognitive rumination) and failure to adapt (issues with sleep, concentration, and functionality). It shows evidence of adequate reliability and validity. Specifically, the internal consistency reliability of the ADNM-8 measured with the Cronbach alpha for the total ADNM-8 scale was high (α=0.83) and also for the preoccupation (α=0.85), and the failure to adapt (α=0.7) subscales (52).

##### Clinical Global Impression Scale (CGI)

This is a Spanish-validated scale that qualitatively describes the severity of the condition and the change observed in the patient compared to the baseline state (50). The subscale evaluating improvement due to treatment was used to ensure construct stability. Improvement is defined as the distance between the patient’s current condition and the condition recorded at the beginning of treatment. This subscale item was completed by both the patient and the evaluator who administered the scale during the initial assessment.

#### Statistical analysis

The sociodemographic and clinical characteristics of the participants were described using descriptive statistics. For quantitative variables, the mean and standard deviation (SD) were presented if they had a normal distribution; otherwise, the median and interquartile range were used. Qualitative variables were reported using absolute and relative frequencies. For the description of stress events, a quantitative analysis of textual data was conducted with natural language processing to calculate the frequency of words written by the patients. The frequency of response options for each item, missing values, and the presence of floor and ceiling effects (defined as values exceeding 15%) were also assessed (46). Missing values in the items were imputed using the mean (simple imputation) due to their low frequency (55).

For structural validity, the Kaiser-Meyer-Olkin (KMO) test was conducted to assess the suitability of the data for factor analysis, with values considered acceptable if greater than 0.70 (56). Next, the number of dimensions was examined using Horn’s parallel analysis, which contrasts observed eigenvalues with expected eigenvalues through resampling techniques (57). Based on the suggested number of factors, exploratory factor analysis (EFA) was performed using principal axis factoring with a polychoric correlation matrix and oblique rotation to accommodate potentially correlated dimensions. Principal axis factoring represents observed correlations through a latent variable, is unaffected by violations of normality assumptions, and is robust to unequal factor loadings or few items per factor (58). Factor loadings were evaluated. Additionally, McDonald’s omega, with its corresponding confidence interval (CI95%), and Cronbach’s alpha were calculated for each identified dimension to assess internal consistency.

The discrimination (a) and difficulty (b) parameters were also analyzed using Item Response Theory (IRT). Parameters were estimated using a generalized partial credit model for polytomous items (45) applied to each dimension according to the solution derived from factor analysis to ensure the assumption of unidimensionality (50). The a parameter, also known as the slope, measures the strength of the relationship between the item and the latent variable (in this case, the AD); the b parameter, or threshold parameters, represent the points along the latent variable where the item response categories are most informative (59). The fit was evaluated for each item based on the values of the infit and outfit statistics, which were considered adequate with values >0.4 and <1.6 (29). Additionally, category response curves (CRC) were constructed for each item.

Test-retest reliability was assessed using the ICC for absolute agreement (95% CI) for the entire scale and each dimension, with a value of ≥0.70 considered adequate (46). For concurrent criterion validity, the dichotomous diagnosis of AD as determined by the independent liaison psychiatrist was used as the reference standard. To consider the present AD, all the symptoms on the checklist should be verified. The area under the receiver operating characteristic curve (AUROC) was calculated, and based on this curve, a cutoff point was established to compute sensitivity, specificity, positive predictive value (PPV), negative predictive value (NPV), and likelihood ratios.

Evidence of convergent construct validity was obtained by calculating Spearman’s rank correlation coefficient for scores obtained on the HADS and the ADNM-8. *A priori*, it was hypothesized that moderate to strong positive correlations (60), with correlation coefficients greater than 0.60 would be found, as these measures assess related constructs. It was also hypothesized that patients diagnosed with AD would score higher on the ETAM compared to those without AD, with significant differences and a moderate effect size, as determined by calculating Hedges’ g for the mean differences between two independent samples (61). Although the instruments were previously validated, we also measured the internal consistency of the HADS and the ADNM-8 scales with Cronbach’s alpha to ensure that their reliability is maintained in our sample.

Data were recorded in REDCap (62), and statistical analysis was conducted using R software (63) and R Studio (64), with the packages: ‘psych’ (and its functions for factor and reliability analysis) (65), ‘quanteda’ (66), MBESS (67), and ‘ltm’ (for IRT modeling) (68).

## Results

### Items and scale development

The operational definition proposed by the SDG centers on the presence of a depressive or anxious syndrome attributed to a stressful event, which generates dysfunction and is considered by the patient as disproportionate (**Table 1**). In alignment with this, the conceptual domains were: 1) depressive and anxious symptoms related to the medical condition; 2) attribution to stressful events; 3) perception of disproportion; and 4) dysfunctionality. This syndrome may meet the criteria for a major depressive episode, as it was considered to be in the same hierarchy and not merely a diagnosis of exclusion, and it is not better explained by substance effects or another clinical condition. Using qualitative research, SDG meetings, and in-depth interviews, a pool of 64 self-report items was created, of which 31 items were approved by the SDG to form the first version of the scale.

**Table 1:** Operational definition of adjustment disorder developed by the Scale Development Group.

COMPONENT	DEFINITION
Mental syndrome	Depressive and anxious syndrome that includes emotional, cognitive, behavioral, and physical manifestations…
Attribution to a stressful event	Attributed to a perceived stressful event…
	*Stressful event: Defined as a situation (whether acute or persistent) that disrupts the course of daily life and is experienced as a demand or change necessary to maintain previous functioning. This can occur, for example, in the following areas:* * • Physical or mental health (personal or family) or its care process* *• Work, study, or personal finances* *• Interpersonal relationships* *• The political environment and violence*
Specific presentation mode	Characterized by high variability in the clinical course, with fluctuations in symptom intensity due to the strong dependence on the stressor or its consequences, as well as the ability to modulate symptoms…
Perception of disproportionality	Which represents a way of reacting to the event that is perceived as disproportionate by the individual themselves, to the point of feeling overwhelmed, and where attempts to cope with the event or its consequences have not been effective…
Dysfunction	And leads to experiencing interference with daily functioning and/or significant distress, including the perception of needing help to cope with the stressor.
This syndrome may meet the criteria for a major depressive episode and is not better explained by the effects of substances or another clinical condition. The attribution to the event established with the patient is the criterion used, not the chronological relationship between the occurrence and the onset of the syndrome. The syndrome resolves with the cessation of the stressor or when the person perceives themselves as adapted.	

Five rounds of cognitive interviews, each with seven patients (n=35), were necessary. These patients were men (n=18; 51.4%) and women (n=17; 48.6%) ranging in age from 42 to 58 years, with diseases of medical (n=21; 60.0%) and surgical (n=14; 40.0%) origin. During the cognitive interviews, it was observed that having a predefined list of stressful situations categorized by themes (health, economy, social relationships, political and social situation) made it difficult for patients to classify their stressful situation or led them to seek situations related to the theme, even if they were not stressful. As a result, it was decided to allow patients to freely describe the situation they were experiencing rather than providing predefined options, as the conceptual emphasis is on their reaction to these situations.

Following this process, a preliminary version of the scale with 22 items was developed, which showed adequate content validity indices, with high agreement among evaluators (**Table 2**). Thus, the Adjustment Disorder Scale for Medically Ill Patients (ETAM, from the Spanish “***E*** *scala del* ***T*** *rastorno de* ***A*** *daptación en pacientes* ***M*** *édicamente enfermos”)* was finalized and approved by the SDG. Conceptually, the scale addresses the free description of perceived stressful situations that occurred in the past 15 days and then asks about affective, cognitive, behavioral, and physical symptoms, which are assessed on how frequently (1=Never, 2=Rarely, 3=Frequently, 4=Always) the subject has experienced them in the past 15 days.

**Table 2:** Content validity assessment of the 22 initial items of the Adjustment Disorder Scale for Medically Ill Patients (ETAM) by 12 experts.

ITEM	JUDGE												CVI-I
	1	2	3	4	5	6	7	8	9	10	11	12	
1. You have felt sad about these stressful situations	4	4	4	4	4	4	4	4	4	4	4	4	1.00
2. Because of these stressful situations, you have felt like crying.	4	4	4	4	4	4	4	4	2	4	3	4	0.92
3. Because of these stressful situations you have felt anxious.	4	4	4	4	4	4	4	4	4	4	4	4	1.00
4. Due to these stressful situations you have been irritable or short-tempered.	4	4	4	4	4	4	4	4	4	4	3	4	1.00
5. You have been afraid of what might happen in these stressful situations	4	4	4	4	4	4	4	4	4	4	4	4	1.00
6. You have been turning these stressful situations over and over in your mind	4	4	4	4	4	4	4	4	4	4	4	4	1.00
7. You have wished to die so as not to have to live these stressful situations	4	4	4	4	4	4	3	4	4	4	4	4	1.00
8. Because of these stressful situations you have thought of committing suicide	4	4	4	4	4	4	3	4	4	4	4	4	1.00
9. Because of these stressful situations, you have thought that you no longer have reasons to live.	4	4	4	4	4	4	4	4	4	4	4	4	1.00
10. Because of these stressful situations, you have had trouble sleeping	4	4	4	4	4	4	2	4	4	4	4	4	0.92
*11. You have felt guilty because of these stressful situations* *	4	1	4	4	4	4	3	4	3	4	3	4	0.92
12. Because of these stressful situations, your appetite has changed.	4	4	4	4	4	4	1	4	2	4	4	4	0.83
13. Because of these stressful situations, you consume more alcohol, cigarettes or drugs than before.	4	4	4	4	4	4	3	4	4	4	4	4	1.00
14. Because of these stressful situations you are less interested in following the treatments for your disease	4	4	4	4	4	4	4	4	4	4	3	4	1.00
15. It is difficult to control your emotional reactions to these stressful situations	4	4	4	4	4	4	4	4	4	4	4	4	1.00
16. The cause of your emotional discomfort is these stressful situations.	4	4	4	4	4	2	4	4	3	4	4	4	0.92
17. Your emotional reactions to these stressful situations are more intense than what is normal for you.	4	4	4	4	4	4	3	4	4	4	4	4	1.00
18. You feel that you have been defeated by these stressful situations	4	4	4	4	4	4	3	4	4	4	4	4	1.00
19. You feel that you need psychological help to cope with these stressful situations	4	1	4	4	4	4	2	4	4	4	4	4	0.83
20.You believe that you can do something to handle these stressful situations*	4	1	4	4	4	4	4	4	2	4	4	4	0.83
21. Because of these stressful situations, you have had problems in your relationship with other people	4	4	4	4	4	4	2	4	4	4	4	4	0.92
22. Because of these stressful situations, you are less interested in taking care of your health.	4	2	4	4	4	4	3	4	4	4	4	4	0.92
**Comprehensiveness of the scale**	4	4	4	4	4	3	4	4	4	3	3	4	1.00
S-CVI/Ave = 0.95													
Percentage of agreement: 91.32%; Fleiss Kappa = 0.83 (95%CI 0.73 - 0.92)													

* This item was removed from the final version after analyzing the scale structure but is shown to maintain item numbering in the analyses presented in this manuscript.

CVI-I, Content Validity Index for the level of each item; S-CVI/Ave, Content Validity Index at the scale level, average type.

The scale also assesses the attribution of symptoms to stressful events, the disproportion of the reaction, and perceived dysfunction through Likert-type questions (1=Strongly Disagree, 2=Disagree, 3=Agree, and 4=Strongly Agree). The item scores were conceived as the raw sum of responses, with a minimum score of 20 and a maximum score of 80. In the pilot test conducted (n=20), no missing items were found, no difficulties with administration were reported, and the average time to complete the scale was 7 minutes and 15 seconds (median of 6 minutes and 22 seconds).

### Participants and their responses

A total of 512 patients were included in the validation phase of the study. The participants were primarily in their fourth and sixth decades of life, with educational backgrounds ranging from primary to secondary schooling. The majority resided in urban areas, were of Catholic faith, and represented both public and private health insurance schemes. The majority were evaluated during hospitalization for various medical conditions, with internal medicine and general surgery, including their sub-specialties, being the most frequently treating specialties (**Table 3**). 87.5% of participants had no history of mental disorders, and 71.7% had no history of substance use.

**Table 3:** Sociodemographic and clinical characteristics of patients assessed with the Adjustment Disorder Scale for Medically Ill Patients (ETAM) (n=512).

CHARACTERISTIC		
Male, n (%)	267	(52.1)
Age, Me (IQR)	56	(39 – 66)
Marital status, n (%)		
Single	171	(33.4)
Married	139	(27.2)
Common-law partnership	118	(23.0)
Separated/Divorced	43	(8.4)
Widowed	41	(8.0)
Education level, n (%)		
None	44	(8.6)
Primary	193	(37.7)
Secondary	178	(34.8)
Technical/Technological	59	(11.5)
Bachelor's	32	(6.2)
Master's	6	(1.2)
Occupation		
Employed	91	(17.8)
Self-employed	149	(29.1)
Unemployed	49	(9.6)
Out of the labor force	28	(5.5)
Student	9	(1.7)
Housewife	132	(25.8)
Retired	54	(10.5)
Social Security		
Contributory	245	(47.8)
Subsidized	242	(47.3)
Other	25	(4.9)
Urban residence, n (%)	357	(69.7)
Religion		
Catholic	385	(75.2)
Christian	53	(10.4)
Others	37	(7.2)
None	37	(7.2)
Evaluation setting		
Hospital	476	(93.0)
Outpatient	36	(7.0
Primary diagnosis (CIE-10 category)		
Infectious diseases	22	(4.3)
Neoplasms	71	(13.9)
Blood and immune	32	(6.3)
Endocrine, nutritional and metabolic	21	(4.1)
Nervous system	14	(2.7)
Eye, ear and mastoid process	3	(0.6)
Circulatory system	61	(11.9)
Respiratory system	16	(3.1)
Digestive system	47	(9.2)
Skin and subcutaneous tissue	17	(3.3)
Musculoskeletal and connective tissue	31	(6.1)
Genitourinary	22	(4.3)
Pregnancy, childbirth and the puerperium	1	(0.2)
Injury/poison	113	(22.1)
Other	41	(8.0)
Treating specialty		
Allergology	2	(0.4)
Cardiology	14	(2.7)
General Surgery and Subspecialties	121	(23.6)
Plastic Surgery	24	(4.7)
Dermatology	1	(0.2)
Endocrinology	9	(1.8)
Gastroenterology	4	(0.8)
Geriatrics	1	(0.2)
Gynecology	16	(3.1)
Hematology-Oncology	37	(7.2)
Infectious Diseases	4	(0.8)
Internal Medicine	112	(21.8)
Nephrology	9	(1.8)
Pulmonology	4	(0.8)
Neurosurgery	14	(2.7)
Neurology	20	(3.9)
Ophthalmology	1	(0.2)
Orthopedics	91	(17.7)
Otorhinolaryngology	8	(1.6)
Rheumatology	8	(1.6)
Toxicology	6	(1.2)
Urology	6	(1.2)
History of mental disorders, n (%)		
None	448	(87.5)
Depressive disorders	28	(5.4)
Anxiety disorders	27	(5.3)
Bipolar disorder	5	(1.0)
Stress disorders	2	(0.4)
Personality disorder	2	(0.4)
Substance use*		
None	367	(71.7)
Alcohol	85	(16.6)
Cigarettes	96	(18.8)
Cannabis	28	(5.5)
Cocaine	15	(2.9)
Opioids	1	(0.2)

*Substances used are not mutually exclusive.

Me, Median; IQR, Interquartile range.

Regarding the stressors reported by patients, health-related issues were the most frequent, but economic and family problems were also mentioned, and 10.9% did not report any stressors (**Table 4**). The frequency of responses (**Table 5**) shows missing values of less than 0.4% and a floor effect indicated by over 80% of responses being “Never” for items 7 (“You have wished to die so as not to have to live these stressful situations?”), 8 (“Because of these stressful situations you have thought of committing suicide”), and 9 (“Because of these stressful situations, you have thought that you no longer have reasons to live”). Given their importance, these items were included in the final version.

**Table 4:** Perceived stressful events and words used by patients assessed with the Adjustment Disorder Scale for Medically Ill Patients (ETAM) (n=512).

Type of stressor	Frequency, n (%)	
Health-related and care-related stressors*	425	83.0
"Illness"	108	21.1
"Hospitalization" and related terms	107	20.9
"Pain"	85	16.6
"Surgery" and "procedure"	33	6.4
"Being away"	16	3.1
"Diagnosis"	10	2.0
"Health"	11	2.1
"Treatment"	11	2.1
"Chemotherapy"	11	2.1
"Accident"	7	1.4
"Cancer"	7	1.4
"Burn"	7	1.4
"Amputation"	7	1.4
"Illness"	5	0.9
Economic problems*	68	13.3
"Economic situation"	37	7.2
"Work"	21	4.1
"Money", "cash"	10	2.0
Family problems*	151	29.5
"Family", "relatives"	61	11.9
"Children"	48	9.4
"Loneliness"	29	5.7
"Being alone"	11	2.1
"Separation"	2	0.4
**None ("Nothing")**	56	10.9

*Not mutually exclusive.Shown in bold are the categories of stressful events perceived by patients and below are the literal words used by them.

**Table 5:** Frequency of responses to the 22 initial items of the Adjustment Disorder Scale for Medically Ill Patients (ETAM) (n=512).

Item	Response Options	Frequency, n (%)		Missing Values
1. You have felt sad about these stressful situations	1 Never	109	(21.3)	0 (0.0%)
	2 Rarely	175	(34.2)	
	3 Frequently	158	(30.9)	
	4 Always	70	(13.7)	
2. Because of these stressful situations, you have felt like crying.	1 Never	145	(28.5)	0 (0.0%)
	2 Rarely	171	(33.4)	
	3 Frequently	157	(30.7)	
	4 Always	38	(7.4)	
3. Because of these stressful situations you have felt anxious.	1 Never	142	(27.8)	1 (0.2%)
	2 Rarely	158	(30.9)	
	3 Frequently	150	(29.4)	
	4 Always	61	(11.9)	
4. Due to these stressful situations you have been irritable or short-tempered.	1 Never	253	(49.4)	0 (0.0%)
	2 Rarely	156	(305)	
	3 Frequently	81	(15.8)	
	4 Always	22	(4.3)	
5. You have been afraid of what might happen in these stressful situations.	1 Never	175	(34.4)	4 (0.1%)
	2 Rarely	129	(25.4)	
	3 Frequently	143	(28.1)	
	4 Always	61	(12.0)	
6. You have been turning these stressful situations over and over in your mind.	1 Never	163	(32.0)	2 (0.1%)
	2 Rarely	117	(22.9)	
	3 Frequently	160	(31.4)	
	4 Always	70	(13.7)	
7. You have wished to die so as not to have to live these stressful situations.	1 Never	395	(86.7)	1 (0.2%)
	2 Rarely	62	(12.1)	
	3 Frequently	42	(8.2)	
	4 Always	12	(2.3)	
8. Because of these stressful situations you have thought of committing suicide.	1 Never	443	(86.7)	1 (0.2%)
	2 Rarely	54	(10.6)	
	3 Frequently	12	(2.3)	
	4 Always	2	(0.4)	
9. Because of these stressful situations, you have thought that you no longer have reasons to live	1 Never	418	(81.8)	1 (0.2%)
	2 Rarely	61	(11.9)	
	3 Frequently	24	(4.7)	
	4 Always	8	(1.6)	
10. Because of these stressful situations, you have had trouble sleeping.	1 Never	181	(35.4)	0 (0.0%)
	2 Rarely	179	(35.0)	
	3 Frequently	110	(21.5)	
	4 Always	42	(8.2)	
*11.You have felt guilty because of these stressful situations* *	1 Never	374	(73.0)	0 (0.0%)
	2 Rarely	78	(15.2)	
	3 Frequently	48	(9.4)	
	4 Always	12	(2.3)	
12. Because of these stressful situations, your appetite has changed.	1 Strongly disagree	108	(21.1)	1 (0.2%)
	2 Disagree	159	(31.1)	
	3 Agree	179	(35.0)	
	4 Strongly agree	65	(12.7)	
13. Because of these stressful situations, you consume more alcohol, cigarettes or drugs than before.	1 Strongly disagree	218	(42.7)	2 (0.4%)
	2 Disagree	251	(49.2)	
	3 Agree	30	(5.9)	
	4 Strongly agree	11	(2.2)	
14. Because of these stressful situations you are less interested in following the treatments for your disease.	1 Strongly disagree	209	(40.9)	1 (0.2%)
	2 Disagree	262	(51.3)	
	3 Agree	34	(6.7)	
	4 Strongly agree	6	(1.2)	
15. It is difficult to control your emotional reactions to these stressful situations.	1 Strongly disagree	108	(21.1)	1 (0.2%)
	2 Disagree	233	(45.6)	
	3 Agree	134	(26.2)	
	4 Strongly agree	36	(7.0)	
16. The cause of your emotional discomfort is these stressful situations.	1 Strongly disagree	79	(15.5)	2 (0.4%)
	2 Disagree	63	(12.4)	
	3 Agree	291	(57.1)	
	4 Strongly agree	77	(15.1)	
17. Your emotional reactions to these stressful situations are more intense than what is normal for you.	1 Strongly disagree	111	(21.7)	1 (0.2%)
	2 Disagree	226	(44.2)	
	3 Agree	125	(24.5)	
	4 Strongly agree	49	(9.6)	
18. You feel that you have been defeated by these stressful situations.	1 Strongly disagree	152	(29.7)	1 (0.2%)
	2 Disagree	252	(49.3)	
	3 Agree	88	(17.2)	
	4 Strongly agree	19	(3.7)	
19 You feel that you need psychological help to cope with these stressful situations	1 Strongly disagree	116	(22.7)	1 (0.2%)
	2 Disagree	213	(41.7)	
	3 Agree	148	(29.0)	
	4 Strongly agree	34	(6.7)	
*20. You believe that you can do something to handle these stressful situations* *	1 Strongly disagree	17	(3.3)	3 (0.6%)
	2 Disagree	57	(11.2)	
	3 Agree	330	(64.8)	
	4 Strongly agree	105	(20.6)	
21. Because of these stressful situations, you have had problems in your relationship with other people	1 Strongly disagree	159	(31.2)	2 (0.4%)
	2 Disagree	261	(51.2)	
	3 Agree	79	(15.5)	
	4 Strongly agree	11	(2.2)	
22. Because of these stressful situations, you are less interested in taking care of your health.	1 Strongly disagree	205	(40.1)	1 (0.2%)
	2 Disagree	265	(51.9)	
	3 Agree	39	(7.6)	
	4 Strongly agree	2	(0.4)	

*This item was removed from the final version after analyzing the scale structure but is shown to maintain item numbering in the analyses presented in this manuscript.

### Scale structure

**Figure 1:**
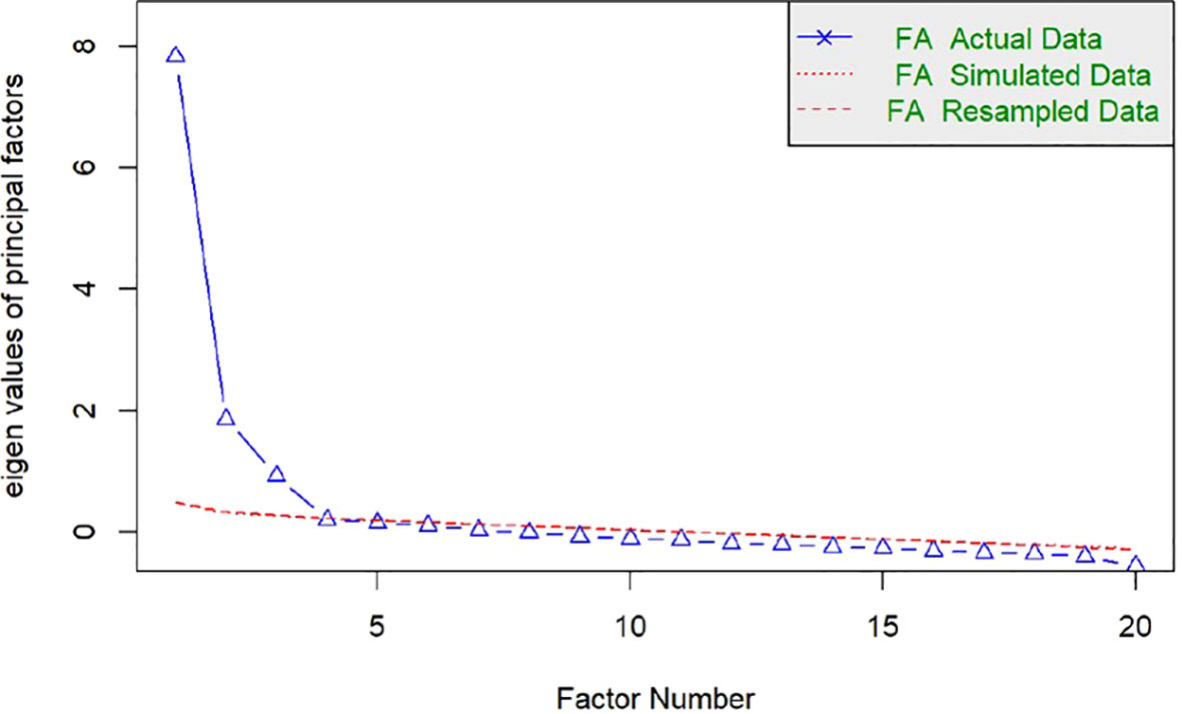
Sedimentation plot from Horn's parallel analysis. The parallel analysis suggests that the number of factors is equal to 3.

The Kaiser-Meyer-Olkin (KMO) measure of sampling adequacy was 0.93. Using a polychoric correlation matrix, correlations ranged from -0.07 to 0.83, suggesting a multidimensional structure (**Table 6**). The Horn’s parallel analysis indicates a three-factor structure (**Figure 1**). In the initial Exploratory Factor Analysis (EFA) with oblique rotation, a three-factor structure was found explaining 67% of the variance.

**Table 6:** Polychoric correlation matrix of the 22 items of the Adjustment Disorder Scale for Medically Ill Patients (ETAM).

	Item1	tem2	Item3	Item4	Item5	Item6	Item7	Item8	Item9	Item10	Item11	Item12	Item13	Item14	Item15	Item16	Item17	Item18	Item19	Item20	Item21	Item22
**Item1**	1.00	0.83	0.73	0.48	0.64	0.69	0.60	0.46	0.59	0.65	0.47	0.53	0.13	0.24	0.54	0.64	0.61	0.48	0.54	0.38	0.39	0.28
**Item2**	0.83	1.00	0.70	0.46	0.65	0.61	0.57	0.50	0.54	0.59	0.46	0.43	0.11	0.19	0.56	0.52	0.56	0.48	0.54	0.41	0.35	0.21
**Item3**	0.73	0.70	1.00	0.47	0.74	0.78	0.56	0.45	0.54	0.59	0.49	0.44	0.04	0.08	0.55	0.59	0.57	0.41	0.47	0.32	0.31	0.05
**Item4**	0.48	0.46	0.47	1.00	0.44	0.49	0.47	0.45	0.43	0.41	0.35	0.34	0.12	0.21	0.47	0.39	0.48	0.30	0.35	0.33	0.44	0.21
**Item5**	0.64	0.65	0.74	0.45	1.00	0.79	0.59	0.51	0.55	0.53	0.47	0.47	-0.07	0.01	0.50	0.52	0.49	0.38	0.39	0.34	0.23	0.02
**Item6**	0.69	0.61	0.78	0.49	0.79	1.00	0.63	0.52	0.57	0.59	0.51	0.51	-0.01	0.07	0.55	0.56	0.58	0.41	0.45	0.32	0.29	0.11
**Item7**	0.61	0.57	0.56	0.47	0.59	0.63	1.00	0.85	0.85	0.59	0.49	0.43	0.28	0.39	0.49	0.51	0.60	0.59	0.50	0.41	0.42	0.40
**Item8**	0.46	0.50	0.45	0.45	0.51	0.52	0.85	1.00	0.84	0.46	0.49	0.34	0.27	0.32	0.41	0.44	0.51	0.49	0.51	0.38	0.39	0.33
**Item9**	0.59	0.54	0.54	0.43	0.55	0.57	0.85	0.84	1.00	0.55	0.39	0.42	0.25	0.32	0.49	0.52	0.58	0.51	0.51	0.41	0.39	0.36
**Item10**	0.65	0.59	0.59	0.41	0.53	0.59	0.59	0.46	0.55	1.00	0.47	0.49	0.11	0.25	0.51	0.52	0.59	0.51	0.48	0.34	0.34	0.31
**Item11**	0.47	0.46	0.49	0.35	0.47	0.51	0.49	0.49	0.39	0.48	1.00	0.26	0.12	0.12	0.43	0.39	0.44	0.29	0.40	0.24	0.29	0.11
**Item12**	0.53	0.43	0.44	0.34	0.47	0.51	0.43	0.34	0.42	0.49	0.26	1.00	0.27	0.38	0.53	0.58	0.59	0.53	0.46	0.41	0.44	0.42
**Item13**	0.13	0.11	0.04	0.12	-0.07	-0.01	0.28	0.27	0.25	0.11	0.12	0.27	1.00	0.63	0.32	0.33	0.35	0.48	0.37	0.37	0.52	0.59
**Item14**	0.25	0.19	0.08	0.21	0.01	0.07	0.39	0.32	0.32	0.25	0.12	0.38	0.63	1.00	0.49	0.38	0.49	0.65	0.41	0.47	0.59	0.81
**Item15**	0.55	0.56	0.55	0.47	0.50	0.55	0.49	0.41	0.49	0.51	0.43	0.53	0.32	0.49	1.00	0.72	0.78	0.64	0.61	0.51	0.55	0.47
**Item16**	0.64	0.52	0.59	0.38	0.52	0.56	0.51	0.44	0.52	0.52	0.39	0.58	0.33	0.38	0.72	1.00	0.75	0.59	0.62	0.36	0.48	0.37
**Item17**	0.61	0.56	0.57	0.48	0.49	0.58	0.60	0.51	0.58	0.59	0.44	0.59	0.35	0.49	0.78	0.75	1.00	0.72	0.65	0.42	0.56	0.52
**Item18**	0.48	0.48	0.41	0.30	0.38	0.41	0.59	0.49	0.51	0.51	0.29	0.53	0.48	0.65	0.64	0.59	0.72	1.00	0.59	0.57	0.55	0.65
**Item19**	0.54	0.54	0.47	0.35	0.39	0.45	0.50	0.51	0.51	0.48	0.40	0.46	0.37	0.41	0.61	0.62	0.65	0.59	1.00	0.34	0.47	0.46
**Item20**	0.38	0.42	0.32	0.33	0.34	0.32	0.41	0.38	0.41	0.34	0.24	0.41	0.37	0.47	0.51	0.36	0.42	0.57	0.34	1.00	0.46	0.43
**Item21**	0.39	0.35	0.31	0.44	0.23	0.29	0.42	0.39	0.39	0.34	0.29	0.44	0.52	0.59	0.55	0.48	0.56	0.55	0.47	0.46	1.00	0.60
**Item22**	0.28	0.21	0.05	0.21	0.02	0.11	0.40	0.33	0.36	0.31	0.11	0.42	0.59	0.81	0.47	0.37	0.52	0.65	0.46	0.43	0.60	1.00

The first factor, labeled “AD Symptoms” (F1), includes items that assess the disproportionate affective symptoms of AD and accounts for 31% of the variance. The second factor, “Impact on Self-Care” (F2), is composed of items addressing the impact on illness-related behavior and explains 18% of the variance. The third factor, “Impact on Desire to Live” (F3), includes items related to death, suicide, and reasons for living. This factor shows a high correlation with the first factor and explains 15% of the variance. This correlation is conceptually plausible since suicidal ideation is part of the symptoms of AD.

**Figure 2:**
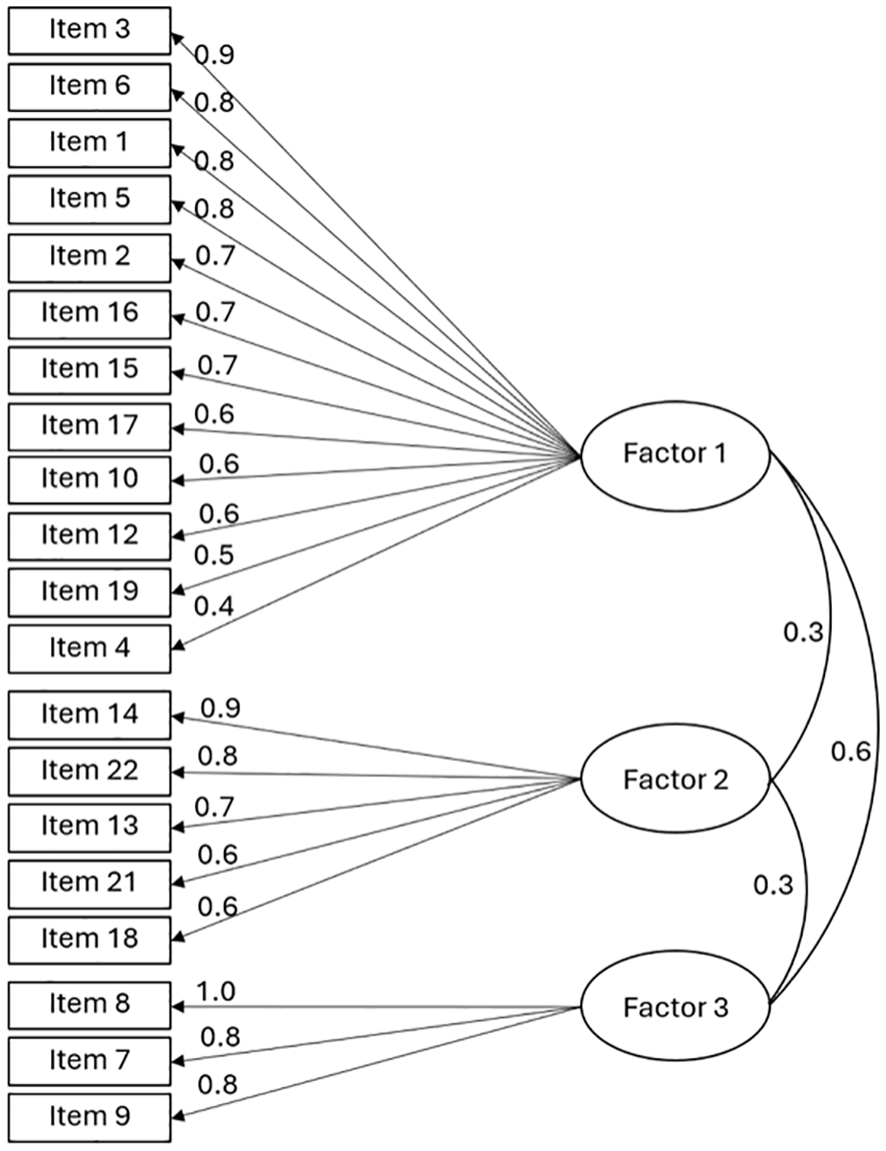
Structure of the final 20-item version of the Adjustment Disorder Scale for Medically Ill Patients (ETAM) from exploratory factor analysis with oblique rotation. Items 11 and 20 were removed.

Upon analyzing the factor loadings of items with this model, it was found that item 11 (“You have felt guilty about these stressful situations”) and item 20 (“You believe that you can do something to handle these stressful situations”) had low factor loadings. It was decided to remove these items because guilt might be a concept present in other disorders, such as depressive disorders, and therefore may not be specific enough; and the belief in the ability to do something, which measured self-efficacy, might be a statement that most people would consider true (as observed in the response frequencies), limiting the item’s usefulness in distinguishing between individuals with different levels of self-efficacy. A new EFA was conducted after excluding these items, which resulted in a similar three-factor configuration (**Figure 2**) explaining 67% of the variance (**Table 7**). This led to the final version of the ETAM with 20 items (**Supplementary Material 1**). It is worth noting that item 19 (“You feel that you need psychological help to cope with these stressful situations”) has a higher loading for F1, consistent with its formulation as part of the assessment of AD dysfunction, i.e., part of its symptoms and therefore was left in this dimension. However, it should not be ignored that it also had a burden for F2, of impact on self-care, which is also clinically consistent because seeking psychological help would be part of self-care.

**Table 7:** Factor loadings for the three-dimensional structure of the Adjustment Disorder Scale for Medically Ill Patients (ETAM) based on exploratory factor analysis without items 11 and 20.

ITEM	FACTOR 1	FACTOR 2	FACTOR 3
Item1	0.79	-0.01	0.09
Item2	0.72	-0.03	0.13
Item3	0.87	-0.19	0.06
Item4	0.43	0.05	0.18
Item5	0.75	-0.28	0.20
Item6	0.79	-0.20	0.17
Item7	0.13	0.07	0.83
Item8	-0.08	0.05	0.96
Item9	0.12	0.05	0.80
Item10	0.59	0.06	0.17
Item12	0.58	0.30	-0.06
Item13	-0.13	0.71	0.11
Item14	-0.06	0.86	0.10
Item15	0.69	0.41	-0.10
Item16	0.71	0.31	-0.06
Item17	0.64	0.42	0.03
Item18	0.35	0.57	0.14
Item19	0.46	0.36	0.11
Item21	0.25	0.58	0.08
Item22	-0.05	0.84	0.13
Eigenvalue	6.48	3.78	3.12
Explained variance	32%	19%	16%
Cumulative explained variance	32%	51%	67%

### Item response theory analysis

**Figure 3:**
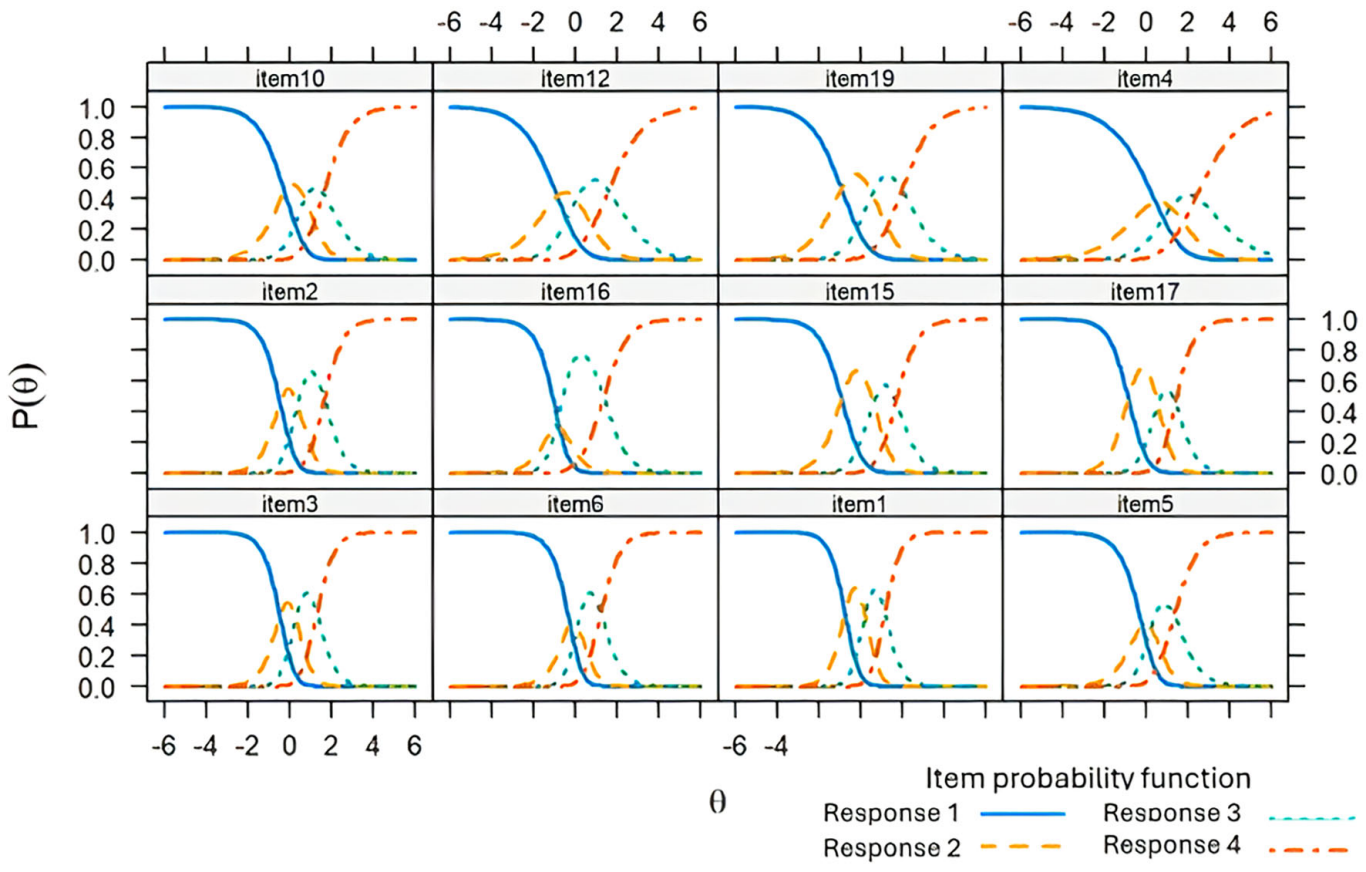
Response category characteristic curve for the items of Dimension 1 of the Adjustment Disorder Scale for Medically Ill Patients (ETAM) using a generalized partial credit model.

**Figure 4:**
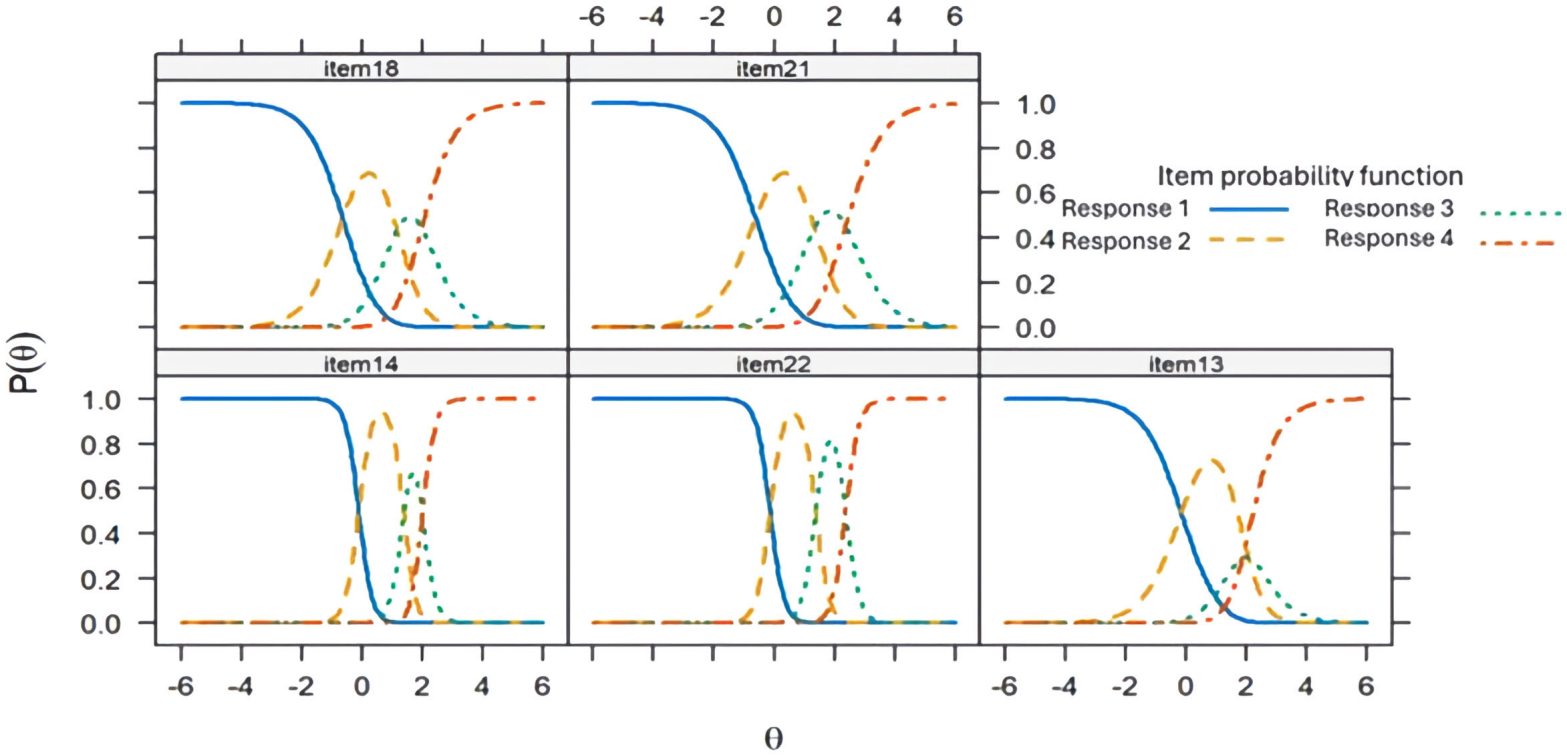
Response category characteristic curve for the items of Dimension 2 of the Adjustment Disorder Scale for Medically Ill Patients (ETAM) using a generalized partial credit model.

**Figure 5:**
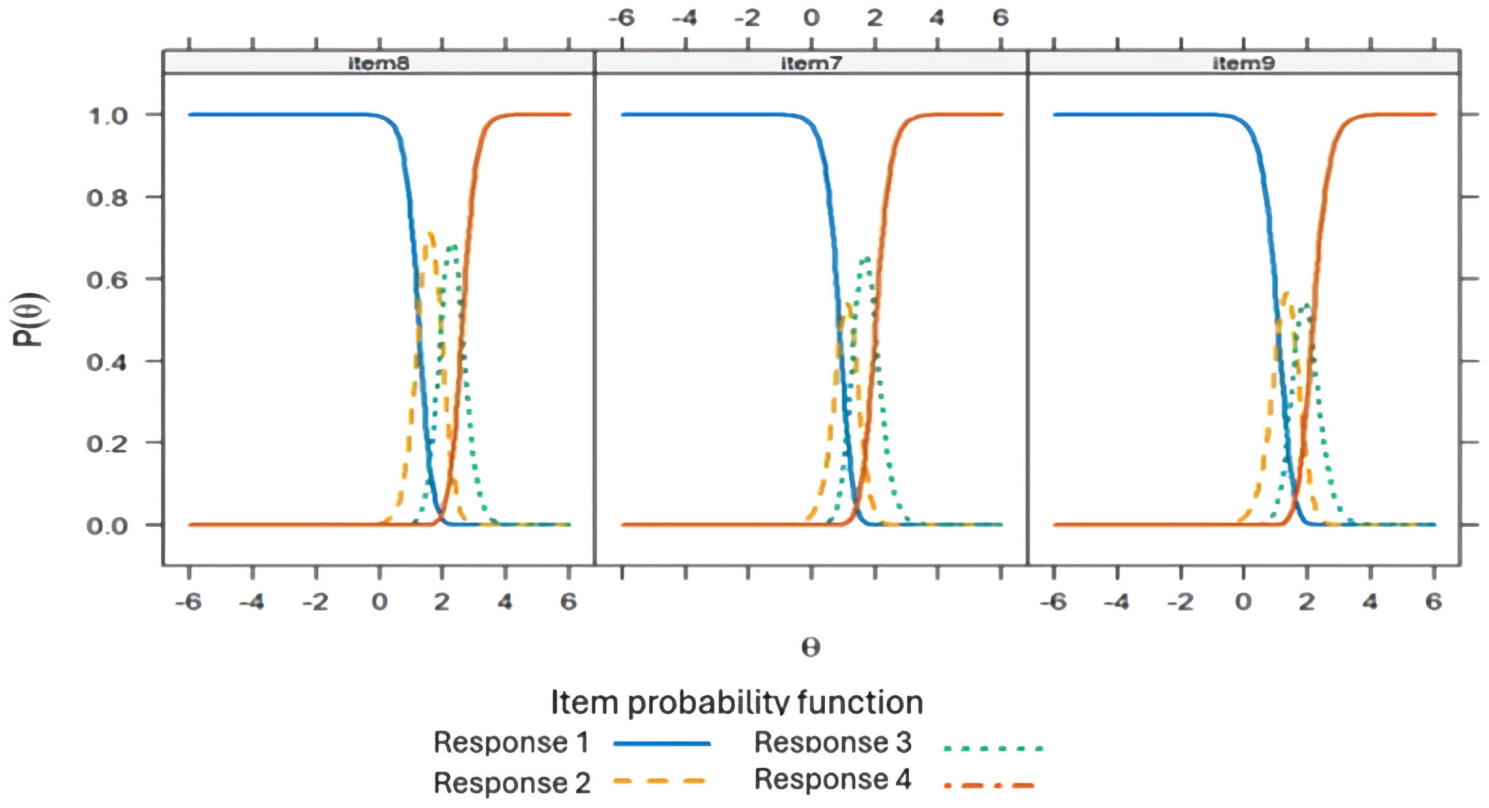
Response category characteristic curve for the items of Dimension 3 of the Adjustment Disorder Scale for Medically Ill Patients (ETAM) using a generalized partial credit model.

The parameters of discrimination and difficulty (**Table 8**). In F1, items with the highest discrimination were item 1 (“You have felt sad about these stressful situations”), item 3 (“Because of these stressful situations you have felt anxious”), item 2 (“Because of these stressful situations, you have felt like crying”), and item 17 (“Your emotional reactions to these stressful situations are more intense than what is normal for you”). In F2, items 14 (“Because of these stressful situations you are less interested in following the treatments for your disease”) and 22 (“Because of these stressful situations, you are less interested in taking care of your health”) showed high discrimination. F3 was observed to be the dimension with the highest discrimination compared to the other dimensions, relating to ideas of death and suicide. The infit was adequate for all items and the outfit was acceptable, except for items 7,8, and 9 in F2. CRC for each item in the three dimensions of the ETAM are presented (**Figures 3**–**5**). Overall, the order of response options was maintained across all items of the scale.

**Table 8:** Discrimination and difficulty parameters of the twenty items* of the Adjustment Disorder Scale for Medically Ill Patients (ETAM), according to the generalized partial credit model of item response theory, and their model fit indexes.

ITEM	a	b1	b2	b3	Infit	Outfit
Factor 1						
Item1	2,61	-0,76	0,21	1,16	0.86	0.78
Item2	2,14	-0,48	0,34	1,64	0.89	0.81
Item3	2,29	-0,49	0,27	1,28	0.86	0.79
Item4	0,87	0,46	1,19	2,39	0.99	0.94
Item5	1,68	0,17	0,23	1,34	0.91	0.84
Item6	2,08	-0,25	0,07	1,21	0.84	0.83
Item10	1,48	-0,27	0,71	1,59	0.94	0.88
Item12	1,10	-0,89	0,03	1,57	0.98	0.96
Item15	1,85	-0,92	0,61	1,68	0.93	0.86
Item16	1,76	-0,78	0,93	1,35	0.94	0.95
Item17	2,09	-0,84	0,57	1,39	0.90	0.83
Item19	1,30	-0,92	0,55	1,98	0.97	0.91
Factor 2						
Item13	1,59	-0,17	1,85	2,05	1.01	0.86
Item14	4,58	-0,11	1,39	2,00	0.79	0.52
Item18	1,67	-0,64	1,16	1,98	0.89	0.88
Item21	1,53	0,61	1,34	2,37	0.92	0.89
Item22	4,42	-0,12	1,38	2,34	0.83	0.53
Factor 3						
Item7	4,03	0,89	1,32	2,01	0.86	0.23
Item8	4,51	1,23	1,95	2,61	0.86	0.21
Item9	3,59	1,08	1,63	2,13	0.92	0.30

a: Discrimination parameter; b: Difficulty or threshold parameters.

* Items 11 and 20 from the original version were removed from the final version of the ETAM.

### Internal consistency

Evidence of adequate internal consistency was found. The McDonald’s Omega coefficient for F1, F2, and F3, it was 0.92 (95%CI: 0.91 – 0.93), 0.83 (95%CI: 0.80 – 0.86), and 0.86 (95%CI: 0.82 – 0.90), respectively. The Cronbach’s alpha for F1 was 0.92 (95%CI: 0.91 – 0.93), for F2 was 0.83 (95%CI: 0.81 – 0.85), and for F3 was 0.84 (95%CI: 0.82 – 0.87).

### Test-retest reliability

A total of 62 patients were evaluated for this property, of which 41 remained stable according to the external criterion of the CGI completed by both the patient and the evaluator. The Intraclass Correlation Coefficient (ICC) between the two measurements was high for the total scale (ICC = 0.98; 95%CI: 0.96 – 0.99), F1 (ICC = 0.99; 95%CI: 0.83 – 1.00), and F3 (ICC = 0.98; 95%CI: 0.97 – 0.99), and adequate for F2 (ICC = 0.69; 95%CI: 0.67 – 0.72).

### Criterion validity

A total of 209 patients were assessed by an independent psychiatrist, with 65 (31.1%) meeting the criteria for a diagnosis of AD. Comparing with the scores of the total scale and its factors, the AUROC values were 0.99 (95%CI: 0.97 – 1.00) for the total score, 0.96 (95%CI: 0.91 – 1.00) for F1, 0.95 (95%CI: 0.91 – 0.99) for F2, and 0.87 (95%CI: 0.79 – 0.94) for F3. For the total score, a cutoff of 43 points or higher was determined, providing a sensitivity of 97.4%, specificity of 90.6%, with 92.9% of patients correctly classified, a positive likelihood ratio of 10.4, and a negative likelihood ratio of 0.02.

### Hypotheses testing for construct Validity

For convergent validity, positive and moderate to high correlations were found between the ETAM and its dimensions with the HADS and the ADNM-8 (**Table 9**). The internal consistency reliability of the anxiety and depression subscales of the HADS in our sample was α=0.74 and α=0.71, respectively. Similarly, the ADNM-8 demonstrated adequate reliability for the preoccupation (α=0.86) and failure to adapt (α=0.82) subscales, as well as for the total scale (α=0.91).

**Table 9:** Convergent construct validity of the final 20-item version of the Adjustment Disorder Scale for Medically Ill Patients (ETAM).

Scale	ETAM			
	Total score	Factor #1	Factor #2	Factor #3
ADNM-8 total	0.86	0.88	0.40	0.69
ADNM-8 preoccupation	0.78	0.81	0.32	0.67
ADNM-8 failure to adapt	0.80	0.82	0.44	0.64
HADS Anxiety	0.68	0.69	0.35	0.55
HADS Depression	0.62	0.61	0.45	0.52

The values presented correspond to the Spearman correlation coefficient.

HADS, Hospital Anxiety and Depression Scale; ADNM-8, 8-item Adjustment Disorder New Module.

Regarding discriminative validity, statistically significant differences were observed in the scores of patients with AD versus those without AD. The average score for patients with AD (n=65) was 54.2 (SD=6.3), whereas for those without AD (n=144), it was 35.1 (SD=6.8), with a large effect size (gHedges=2.75; 95%CI: 2.36 – 3.15).

## Discussion

This study developed a scale for diagnosing and assessing AD in medically ill patients in Colombia and found evidence of its content, structural, reliability, criterion, and convergent and discriminative construct validity. A new theoretical model for AD in medically ill patients was created to address the specific clinical characteristics of AD in this population. Although specific manifestations are added, the developed construct is not opposed to those defined by current diagnostic manuals such as DSM-5-TR (2) and ICD-11 (69), facilitating the unification of criteria, which could lead to a definition that can be systematically applied by mental health professionals in this population.

Evidence of content validity was found for the ETAM, a property seldom reported in diagnostic scale studies and absent in other scales measuring Adjustment Disorder (AD), but crucial for obtaining other validity evidences (70). In fact, during the development of cognitive interviews, we encountered difficulties in presenting patients with a pre-specified list of stressful situations, as is common in other instruments such as the ADNM (71), allowing patients to describe their own stressful experiences without additional cognitive effort is particularly important, considering that nearly 46% of the sample had low or no education levels.

The final 20-item structure of the ETAM revealed that the construct consists of three dimensions. The first dimension encompasses symptoms of AD (F1), the second addresses the impact on self-care (F2), and the third pertains to the impact on the desire to live (F3), all demonstrating adequate internal consistency. Literature typically establishes AD as a unidimensional construct, combining anxious and depressive symptoms as a reaction to a stressful event (72). In the ETAM, Dimension 1 refers to all clinical features of AD, with a high correlation to Dimension 3, which specifically includes thoughts of death and suicide. These symptoms are also captured in current diagnostic manuals (2, 69) and instruments based on them (52). However, the ETAM also includes specific clinical features of AD for the medically ill population, which are grouped in Dimension 2 (F2) “Impact on Self-Care.”

The impact on self-care is a clinically relevant dimension in the medically ill population and refers to manifestations of AD that lead to a decline in self-care. This decline can result in the abandonment of medical treatment and follow-up, leading to worse health outcomes (73), which is why it is important to include it in the conceptualization of AD in these patients. Self-care is understood as the activities an individual initiates independently to maintain health and life, making it an essential component of coping with medical conditions (74). This concept has been operationalized primarily into three components. One external self-care that alludes to actions taken for a particular physical condition. A psychological or internal dimension that includes the mental attitude toward these actions. And a relational dimension reflecting how individuals engage in self-care through interactions with others (75). The ETAM addresses precisely these aspects by evaluating the patient’s commitment to medical treatment, overall health maintenance, substance use, self-perceived defeat, and interpersonal relationship issues related to coping with stressors.

In the external component of self-care, the willingness to engage in positive actions and favorable experiences, such as adhering to medical treatment or maintaining a healthy lifestyle, can be compromised during AD. The interpersonal component, which involves seeking positive interactions with others to meet support and care needs, may be disrupted by emotional reactions to stressors, leading to difficulties in maintaining these interactions and resulting in interpersonal problems. In the internal component, AD can manifest as a loss of the ability to view oneself as a protector and a sense of dejection expressed as defeat. Some studies have indeed found a relationship between affective symptoms and components of self-care. For instance, in patients with heart failure, the relationship between depressive symptoms and self-care maintenance is mediated by self-confidence in self-care, such that more severe depressive symptoms diminish self-confidence in self-care, which in turn reduces the ability to sustain the external component of self-care over time (76).

The perception of defeat included in item 18 (“You feel that you have been defeated by these stressful situations”) of Dimension 2 (F2 - Impact on Self-Care) of the ETAM aligns with this internal component of self-care. The choice of such a term was aimed at maximizing content validity by using the exact words of the medically ill patients interviewed during the item generation phase. As a concept, defeat has been developed within evolutionary theories as a depressogenic event, based on observations from ethological research where socially defeated animals exhibit stress behaviors such as abandoning feeding, social isolation, and autonomic hyperactivity (77). This concept has been extrapolated to humans and beyond social contexts to describe the resulting feeling from failure or loss of life goals. The range of circumstances that can provoke a sense of defeat in humans has expanded beyond direct interpersonal conflict to include other situations perceived as failed struggles, which can lead to depressive and anxious symptoms as well as suicidal behavior (78).

In line with classical theories on human response to stressors, such as Hans Selye’s concept of final exhaustion (79) or Lazarus and Folkman’s theories on coping and appraisal (80, 81), there are dynamic states or phases in the interaction process with a stressor that can lead to unresolved issues, with a loss of coping ability and ongoing adaptation failure. It is possible that in patients with medical conditions, the stress from the disorder can become so intense that it eventually leads to a perception of defeat, a loss of interest in continuing treatments and self-care, and even thoughts of death and suicide. Indeed, when analyzing item response theory, the items related to self-care (F2) and the impact on the desire to live (F3) demonstrated the highest discrimination between individuals with high versus low levels of TA, as well as higher thresholds. These items require a greater level of the latent variable (AD) to be addressed and could provide valuable information for patients formally diagnosed with this condition. Future research could explore the behavior over time of patients with high levels of impact on self-care (F2), including perceptions of defeat, which may indicate different stress levels that predispose them to chronic forms or diagnostic category changes such as MDE. We must also emphasize that the structure found in this study is provisional [or hypothetical (29)] and must be confirmed in an independent sample.

We also conducted an analysis of the ETAM using IRT. One of the key advantages of this methodology is that it enables us to understand the difficulty of the items and the level of the measured trait in individuals, providing valuable insights into the construct being assessed. This approach has become an essential and complementary tool in the validation of scales measuring psychological conditions (82). With IRT, we can determine how much of the AD is required to respond to each item. This allows for selecting items suited to specific purposes and populations. For example, if a clinician aims to screen for AD in the general population, where the level of the trait is expected to be low, they can use the easier items. However, for evaluating the severity or classifying patients with more intense AD, more challenging items should be employed, as the items of F3, that are related to dead and suicide ideas (items 7, 8 and 9). Precisely in these items we found a low outfit, in the presence of good infit, which could be present with very high discrimination, although this index is sensitive to outliers (83).

In this study, we also found that the ETAM had adequate test-retest reliability, with a ICC of 0.98 considered high (84), indicating that the measurement of AD remained stable over a period of 3 to 4 days. The aim of this evaluation is to differentiate between the variance arising from true scores and random, transient measurement error, which can result from fluctuations in patient responses due to information processing or mood changes (85). A short time interval and the use of an external anchor such as the CGI were chosen, given that AD can be fluctuating and demonstrating the stability of the construct can be challenging, which is a primary limitation in psychometric research of this property (86). A longer time interval could have allowed for a true change in the construct; therefore, a short one was used, with the risk that respondents might recall their initial answers (87). However, including the perception of stability from both the clinician and the patient suggests a true stability in the clinical picture rather than mere recall of responses and could enhance confidence in the evidence of the stability over time of the ETAM.

One of the most significant clinical implications of this research is that the ETAM demonstrated a high capacity for accurately classifying patients with and without AD, which could enhance diagnostic practices. As such, it could be used as a screening tool for medically ill patients to facilitate timely diagnosis, enabling comprehensive management with specific psychotherapeutic interventions for each case and rational prescription of psychotropic medications. Indeed, screening for mental health symptoms in medically ill patients has been shown to improve adherence to primary disease treatment and could enhance prognosis (88). However, it is possible that the diagnostic performance observed as evidence of criterion validity might be overestimated, given that the independent clinical evaluation used as the reference test employed the same criteria underpinning the ETAM.

This approach was necessary because the AD model developed by the SDG does not subordinate the diagnosis to meeting criteria for other disorders such as MDE, as outlined in current diagnostic manuals (2, 89). In other words, our conceptualization does not propose AD as a diagnosis of exclusion but as a category encompassing a depressive or anxious syndrome attributable to a stressful event, positioned in a hierarchy comparable to other disorders. Therefore, it would have been inconsistent to use other diagnostic criteria, which could have reduced diagnostic accuracy. Nevertheless, the hypotheses regarding the relationship of the ETAM with other instruments measuring AD in the general population, such as the ADNM-8, and the anxious and depressive symptoms reflected in the HADS, were confirmed. We believe this validates the construct being measured. Furthermore, the alignment of our findings with international literature reinforces the relevance of ETAM as a tool to assess AD in medically ill patients. Studies from Maercker et al. (54) have highlighted the importance of addressing context-specific symptoms of AD, and our work builds on this foundation while addressing gaps specific to medically ill populations. By situating ETAM within this broader research framework, we contribute to expanding the applicability of AD assessments across a specific clinical context.

It is important to highlight that the prevalence of AD found in this study was 31.1%, a value that is high compared to a meta-analysis encompassing 23 studies in oncological and hematological settings, where a prevalence of 19.4% in cancer patients and 15.4% in palliative care settings was reported (90). This finding in our population could be explained by the fact that the studies included in the meta-analysis were conducted in developed countries. It is possible that the Colombian population faces different social determinants unique to a developing country and may therefore be exposed to multiple stressors. Additionally, cultural factors related to concepts of health, illness, and death, timely access to healthcare, as well as life expectations, roles, and functions of individuals in different social contexts, may account for differences in adaptation to stressors (91, 92). Another possible explanation is the non-exclusionary nature of the AD construct developed by the SDG, where AD is not a diagnosis of exclusion. Consequently, cases that might have been labeled as MDE under other criteria could be included.

This study has several limitations that should be acknowledged. Firstly, the ETAM was administered in high-complexity hospitals, and most of the patients were evaluated in an inpatient setting. This context might suggest that the patients have severe medical symptoms, which could lead to an overestimation of health-related concerns. Additionally, the instrument includes items that inquire about behaviors that might be considered socially stigmatized, such as suicidal ideation. This could introduce response biases, with patients potentially minimizing or denying such thoughts to avoid stigma (93). In general, there is reliance on self-reported responses which could introduce in self-reported responses due to social desirability or recall biases. Although self-assessment is the usual method in psychometric validation in mental health (94) and aligns with the patient-centered approach of the ETAM, future studies could integrate clinician-rated scales or objective clinical data to complement self-reported measures and improve the robustness of findings.

Finally, future research should consider evaluating the responsiveness of the ETAM and the longitudinal impact of its implementation to ensure its utility in assessing interventions, as well as its validation in lower-complexity settings such as primary care, where AD is highly prevalent and underlying medical conditions might be less severe (95, 96). Further exploration of clinically significant differences, including comparisons with patients with MDE and gathering more evidence on its discriminative validity, should also be pursued. It should also be noted that the ETAM was developed and validated in a Colombian population, which may limit its applicability in other cultural contexts. Cultural differences can affect the perception, expression, and reporting of AD. As such, further research is needed to adapt and validate the scale for use in other cultural settings. Cross-cultural validation would ensure its relevance and utility in capturing the nuances of adjustment disorder globally.

## Conclusion

This study provided evidence of the validity and reliability of the ETAM in medically ill patients, demonstrating its potential clinical utility for the timely identification of individuals at higher risk of experiencing AD.

## Data Availability

The raw data supporting the conclusions of this article will be made available by the authors, without undue reservation.

## References

[1] Casey P Jabbar F. Adjustment disorder considered. Adv Psychiatr Treat. (2013) 19:99–107. doi: 10.1192/apt.bp.111.010058

[2] American Psychiatric Association. Diagnostic and Statistical Manual of Mental Disorders. Fifth Edition. Washington, DC: American Psychiatric Association Publishing (2022). Text Revision (DSM-5-TR).

[3] Wijnhoven LMA Kwakkenbos L Verdonck-de Leeuw IM Prins JB Custers JAE. Evaluating time-limited and persistent symptoms of adjustment disorder in cancer patients after a colorectal cancer diagnosis: a longitudinal observational study. J Psychosoc Oncol Res Pract. (2023) 5:1–7. doi: 10.1097/OR9.0000000000000105

[4] Stein DJ Rouillon F Maercker A. New perspectives on adjustment disorder. World J Biol Psychiatry. (2018) 19:S1–2. doi: 10.1080/15622975.2018.1433327 30204564

[5] Gradus JL Qin P Lincoln AK Miller M Lawler E Lash TL. The association between adjustment disorder diagnosed at psychiatric treatment facilities and completed suicide. Clin Epidemiol. (2010) 2:23–8. doi: 10.2147/CLEP.S9373 PMC2943177 20865099

[6] Fleischmann A Bertolote JM Belfer M Beautrais A. Completed suicide and psychiatric diagnoses in young people: A critical examination of the evidence. Am J Orthopsychiatry. (2005) 75:676–83. doi: 10.1037/0002-9432.75.4.676 16262523

[7] Marttunen MJ Aro HM Henriksson MM Lönnqvist JK. Adolescent suicides with adjustment disorders or no psychiatric diagnosis. Eur Child Adolesc Psychiatry. (1994) 3:101–10. doi: 10.1007/BF01977672 29871468

[8] Kryzhanovskaya L Canterbury R. Suicidal behavior in patients with adjustment disorders. Crisis. (2001) 22:125–31. doi: 10.1027//0227-5910.22.3.125 11831599

[9] Portzky G Audenaert K van Heeringen K. Adjustment disorder and the course of the suicidal process in adolescents. J Affect Disord. (2005) 87:265–70. doi: 10.1016/j.jad.2005.04.009 16005078

[10] Shaffer D. Psychiatric diagnosis in child and adolescent suicide. Arch Gen Psychiatry. (1996) 53:339. doi: 10.1001/archpsyc.1996.01830040075012 8634012

[11] Catalina-Romero C Pastrana-Jimenez JI Tenas-Lopez MJ Martinez-Munoz P Ruiz-Moraga M Fernandez-Labandera C. Long-term sickness absence due to adjustment disorder. Occup Med (Chic Ill). (2012) 62:375–8. doi: 10.1093/occmed/kqs043 22544846

[12] Chirico F. Adjustment disorder as an occupational disease: our experience in Italy. Int J Occup Environ Med. (2016) 7:52–7. doi: 10.15171/ijoem.2016.716 PMC6816519 26772598

[13] Gradus JL Bozi I Antonsen S Svensson E Lash TL Resick PA. Severe stress and adjustment disorder diagnoses in the population of Denmark. J Trauma Stress. (2014) 27:370–4. doi: 10.1002/jts.21926 PMC7001752 24948539

[14] Hay SI Abajobir AA Abate KH Abbafati C Abbas KM Abd-Allah F. Global, regional, and national disability-adjusted life-years (DALYs) for 333 diseases and injuries and healthy life expectancy (HALE) for 195 countries and territories, 1990–2016: a systematic analysis for the Global Burden of Disease Study 2016. Lancet. (2017) 390:1260–344. doi: 10.1016/S0140-6736(17)32130-X PMC5605707 28919118

[15] Van Beek FE Wijnhoven LMA Custers JAE Holtmaat K De Rooij BH Horevoorts NJE. Adjustment disorder in cancer patients after treatment: prevalence and acceptance of psychological treatment. Support Care Cancer. (2022) 30:1797–806. doi: 10.1007/s00520-021-06530-0 PMC8486632 34599663

[16] Tang H Xiong H Deng L Fang Y Zhang J Meng H. Adjustment disorder in female breast cancer patients: prevalence and its accessory symptoms. Curr Med Sci. (2020) 40:510–7. doi: 10.1007/s11596-020-2205-1 32474858

[17] Olsson KM Meltendorf T Fuge J Kamp JC Park D-H Richter MJ. Prevalence of mental disorders and impact on quality of life in patients with pulmonary arterial hypertension. Front Psychiatry. (2021) 12:667602. doi: 10.3389/fpsyt.2021.667602 34135787 PMC8200462

[18] Princip M Ledermann K Altwegg R von Känel R. Cardiac disease-induced trauma and stress-related disorders. Herz. (2024) 49:254–60. doi: 10.1007/s00059-024-05255-0 PMC11286693 38990256

[19] Shafierizi S Basirat Z Nasiri-Amiri F Kheirkhah F Chehrazi M Pasha H. The prevalence of adjustment disorder and predisposing factors in infertile women. BMC Psychol. (2023) 11:142. doi: 10.1186/s40359-023-01193-4 37131228 PMC10152011

[20] Smith ML Farkas DK Sumner JA Jiang T Lash TL Galea S. Associations between adjustment disorder and hospital-based infections in the Danish population. J Psychosom Res. (2020) 132:109976. doi: 10.1016/j.jpsychores.2020.109976 32142971

[21] Rackley S Bostwick JM. Depression in medically ill patients. Psychiatr Clin North Am. (2012) 35:231–47. doi: 10.1016/j.psc.2011.11.001 22370500

[22] Cavanaugh S von A. Depression in the hospitalized inpatient with various medical illnesses. Psychother Psychosom. (1986) 45:97–104. doi: 10.1159/000287934 3786646

[23] Rodin G Voshart K. Depression in the medically ill: an overview. Am J Psychiatry. (1986) 143:696–705. doi: 10.1176/ajp.143.6.696 3521339

[24] O’Keeffe N Ranjith G. Depression, demoralisation or adjustment disorder? Understanding emotional distress in the severely medically ill. Clin Med (Northfield Il). (2007) 7:478–81. doi: 10.7861/clinmedicine.7-5-478 PMC4953046 17990716

[25] Beal ML Lerman SF Leppla IE. When is being sad on the burn unit pathological? Differential diagnosis of demoralization, adjustment disorder and major depressive disorder in burn survivors. Eur Burn J. (2022) 3:122–34. doi: 10.3390/ebj3010010 PMC11575370 39604179

[26] Olver JS Hopwood MJ. Depression and physical illness. Med J Aust. (2012) 1:9–12. doi: 10.5694/mjao12.10597 25370291

[27] Shevlin M Hyland P Ben-Ezra M Karatzias T Cloitre M Vallières F. Measuring ICD-11 adjustment disorder: the development and initial validation of the International Adjustment Disorder Questionnaire. Acta Psychiatr Scand. (2020) 141:265–74. doi: 10.1111/acps.13126 31721147

[28] Bachem R Perkonigg A Stein DJ Maercker A. Measuring the ICD-11 adjustment disorder concept: Validity and sensitivity to change of the Adjustment Disorder - New Module questionnaire in a clinical intervention study. Int J Methods Psychiatr Res. (2017) 26:e1545. doi: 10.1002/mpr.1545 27862575 PMC6877162

[29] Boateng GO Neilands TB Frongillo EA Melgar-Quiñonez HR Young SL. Best practices for developing and validating scales for health, social, and behavioral research: A primer. Front Public Heal. (2018) 6:149. doi: 10.3389/fpubh.2018.00149 PMC6004510 29942800

[30] Mokkink LB Terwee CB Knol DL Stratford PW Alonso J Patrick DL. The COSMIN checklist for evaluating the methodological quality of studies on measurement properties: a clarification of its content. BMC Med Res Methodol. (2010) 10:22. doi: 10.1186/1471-2288-10-22 20298572 PMC2848183

[31] Berk RA. Importance of expert judgment in content-related validity evidence. West J Nurs Res. (1990) 12:659–71. doi: 10.1177/019394599001200507 2238643

[32] Ricci L Lanfranchi J-B Lemetayer F Rotonda C Guillemin F Coste J. Qualitative methods used to generate questionnaire items: A systematic review. Qual Health Res. (2019) 29:149–56. doi: 10.1177/1049732318783186 29952223

[33] Zapata-Ospina JP Jiménez-Benítez M Fierro M. I was very sad, but not depressed”: phenomenological differences between adjustment disorder and a major depressive episode. Front Psychiatry. (2023) 14:1291659. doi: 10.3389/fpsyt.2023.1291659 38146279 PMC10749326

[34] Zapata-Ospina JP Sierra-Muñoz J Madrid P Yepes-Delgado CE. The adjustment disorder is not a wastebasket diagnosis: a grounded theory study of psychiatrists’ and psychologists’ clinical reasoning. Eur J Psychotraumatol. (2024) 15(1):2390332. doi: 10.1080/20008066.2024.2390332 39166284 PMC11340213

[35] Muñiz J Fonseca-Pedrero E. Diez pasos para la construcción de un test. Psicothema. (2019) 31:7–16. doi: 10.7334/psicothema2018.291 30664405

[36] Kyriazos TA Stalikas A. Applied psychometrics: the steps of scale development and standardization process. Psychology. (2018) 09:2531–60. doi: 10.4236/psych.2018.911145

[37] Leidy NK Vernon M. Perspectives on Patient-Reported Outcomes: content validity and qualitative research in a changing clinical trial environment. Pharmacoeconomics. (2008) 26:363–70. doi: 10.2165/00019053-200826050-00002 18429654

[38] Padilla J-L Benítez I. Validity evidence based on response processes. Psicothema. (2014) 26:136–44. doi: 10.7334/psicothema2013.259 24444741

[39] Peterson CH Peterson NA Powell KG. Cognitive interviewing for item development: validity evidence based on content and response processes. Meas Eval Couns Dev. (2017) 50:217–23. doi: 10.1080/07481756.2017.1339564

[40] Blair J Ackermann A Piccinino L Levenstein R. Using behavior coding to validate cognitive interview findings. Proc Am Stat Assoc Sect. Surv. Res Methods Salt Lake City UT. (2007), 3896–900.

[41] Terwee CB Prinsen CAC Chiarotto A Westerman MJ Patrick DL Alonso J. COSMIN methodology for evaluating the content validity of patient-reported outcome measures: a Delphi study. Qual Life Res. (2018) 27:1159–70. doi: 10.1007/s11136-018-1829-0 PMC5891557 29550964

[42] Polit DF Beck CT Owen SV. Is the CVI an acceptable indicator of content validity? Appraisal and recommendations. Res Nurs Health. (2007) 30:459–67. doi: 10.1002/nur.20199 17654487

[43] Davis LL. Instrument review: Getting the most from a panel of experts. Appl Nurs Res. (1992) 5:194–7. doi: 10.1016/S0897-1897(05)80008-4

[44] Wolf EJ Harrington KM Clark SL Miller MW. Sample size requirements for structural equation models. Educ Psychol Meas. (2013) 73:913–34. doi: 10.1177/0013164413495237 PMC4334479 25705052

[45] de Ayala RJ. The theory and practice of item response theory. New York, United States of America: The Guilford Press (2009).

[46] de Vet HCW Terwee CB Mokkink LB Knol DL. Measurement in Medicine. New York: Cambridge University Press (2011).

[47] Foster P Oxman T. A descriptive study of adjustment disorder diagnoses in general hospital patients. Ir J Psychol Med. (1994) 11:153–7. doi: 10.1017/S0790966700001683

[48] Hanley JA McNeil BJ. The meaning and use of the area under a receiver operating characteristic (ROC) curve. Radiology. (1982) 143:29–36. doi: 10.1148/radiology.143.1.7063747 7063747

[49] Bonett DG Wright TA. Sample size requirements for estimating pearson, kendall and spearman correlations. Psychometrika. (2000) 65:23–8. doi: 10.1007/BF02294183

[50] Casas E Escandell MJ Ribas M Ochoa S. Instrumentos de evaluación en rehabilitación psicosocial. Rev La Asoc Española Neuropsiquiatría. (2010) 30:25–47. doi: 10.4321/S0211-57352010000100002

[51] Tejero Pociello A Guimerá Querol E Farré Martí J Peri J. Uso clínico del HAD (Hospital Anxiety and Depression Scale) en población psiquiátrica: un estudio de su sensibilidad, fiabilidad y validez. Rev Del Dep Psiquiatr La Fac Med Barcelona. (1986) 13:233–8.

[52] Kazlauskas E Gegieckaite G Eimontas J Zelviene P Maercker A. A brief measure of the international classification of diseases-11 adjustment disorder: investigation of psychometric properties in an adult help-seeking sample. Psychopathology. (2018) 51:10–5. doi: 10.1159/000484415 29301130

[53] Terol-Cantero MC Cabrera-Perona V Martín-Aragón M. Revisión de estudios de la Escala de Ansiedad y Depresión Hospitalaria (HAD) en muestras españolas. Psicol. (2015) 31:494. doi: 10.6018/analesps.31.2.172701

[54] Maercker A Einsle F Köllner V. Adjustment disorders as stress response syndromes: A new diagnostic concept and its exploration in a medical sample. Psychopathology. (2007) 40:135–46. doi: 10.1159/000099290 17284941

[55] Streiner D Norman G Cairney J. Health measurement scales. In: A practical guide to their development and use, Fifth Edit. Oxford University Press, New York (2015).

[56] Kaiser HF. An index of factorial simplicity. Psychometrika. (1974) 39:31–6. doi: 10.1007/BF02291575

[57] Horn JL. A rationale and test for the number of factors in factor analysis. Psychometrika. (1965) 30:179–85. doi: 10.1007/BF02289447 14306381

[58] Grieder S Steiner MD. Algorithmic jingle jungle: A comparison of implementations of principal axis factoring and promax rotation in R and SPSS. Behav Res Methods. (2021) 54:54–74. doi: 10.3758/s13428-021-01581-x 34100201 PMC8863761

[59] Stover AM McLeod LD Langer MM Chen W-H Reeve BB. State of the psychometric methods: patient-reported outcome measure development and refinement using item response theory. J Patient-Reported Outcomes. (2019) 3:50. doi: 10.1186/s41687-019-0130-5 PMC6663947 31359210

[60] Cohen J. A power primer. Psychol Bull. (1992) 112:155–9. doi: 10.1037/0033-2909.112.1.155 19565683

[61] Lakens D. Calculating and reporting effect sizes to facilitate cumulative science: a practical primer for t-tests and ANOVAs. Front Psychol. (2013) 4:863. doi: 10.3389/fpsyg.2013.00863 24324449 PMC3840331

[62] Harris PA Taylor R Thielke R Payne J Gonzalez N Conde JG. Research electronic data capture (REDCap)—A metadata-driven methodology and workflow process for providing translational research informatics support. J BioMed Inform. (2009) 42:377–81. doi: 10.1016/j.jbi.2008.08.010 PMC2700030 18929686

[63] R Core Team. R: A Language and Environment for Statistical Computing. Vienna, Austria: R Foundation for Statistical Computing (2023). Available at: https://www.r-project.org (Accessed July 31, 2024).

[64] Studio Team. RStudio: Integrated Development for R. PBC, Boston, MA: RStudio (2020). Available at: http://www.rstudio.com/ (Accessed July 31, 2024).

[65] Revelle W. psych: Procedures for Psychological, Psychometric, and Personality Research. Evanston, Illinois, USA: Northwestern University (2023). Available at: https://cran.r-project.org/package=psych (Accessed July 31, 2024).

[66] Benoit K Watanabe K Wang H Nulty P Obeng A Müller S. quanteda: An R package for the quantitative analysis of textual data. J Open Source Softw. (2018) 3:774. doi: 10.21105/joss.00774

[67] Kelley K. MBESS: The MBESS R Package. R package version 4.9.3. Indiana, USA: University of Notre Dame (2023). Available at: https://cran.r-project.org/package=MBESS (Accessed July 31, 2024).

[68] Rizopoulos D. ltm: an R package for latent variable modeling and item response theory analyses. J Stat Softw. (2006) 17:1–25. doi: 10.18637/jss.v017.i05

[69] World Health Organization (WHO). International Classification of Diseases Eleventh Revision (ICD-11). Geneva: The WHO (2022).

[70] Zapata-Ospina JP García-Valencia J. Validity based on content: A challenge in health measurement scales. J Health Psychol. (2022) 27:481–93. doi: 10.1177/1359105320953477 32945184

[71] Lorenz L Bachem R Maercker A. The adjustment disorder–new module 20 as a screening instrument: cluster analysis and cut-off values. Int J Occup Environ Med. (2016) 7:215–20. doi: 10.15171/ijoem.2016.775 PMC6817961 27651082

[72] Lorenz L Hyland P Perkonigg A Maercker A. Is adjustment disorder unidimensional or multidimensional? Implications for ICD-11. Int J Methods Psychiatr Res. (2018) 27(1):e1591. doi: 10.1002/mpr.1591 28990345 PMC6877110

[73] Alqahtani J Alqahtani I. Self-care in the older adult population with chronic disease: concept analysis. Heliyon. (2022) 8:e09991. doi: 10.1016/j.heliyon.2022.e09991 35874086 PMC9304718

[74] Riegel B Jaarsma T Strömberg A. A middle-range theory of self-care of chronic illness. Adv Nurs Sci. (2012) 35:194–204. doi: 10.1097/ANS.0b013e318261b1ba 22739426

[75] González-Vazquez AI Mosquera-Barral D Knipe J Leeds AM Santed-German MA. Construction and initial validation of a scale to evaluate self-care patterns. Clin Neuropsychiatry. (2018) 15:373–8.

[76] Chang L-Y Wu S-Y Chiang C-E Tsai P-S. Depression and self-care maintenance in patients with heart failure: A moderated mediation model of self-care confidence and resilience. Eur J Cardiovasc Nurs. (2017) 16:435–43. doi: 10.1177/1474515116687179 28059552

[77] Hollis F Kabbaj M. Social defeat as an animal model for depression. ILAR J. (2014) 55:221–32. doi: 10.1093/ilar/ilu002 25225302

[78] Taylor PJ Gooding P Wood AM Tarrier N. The role of defeat and entrapment in depression, anxiety, and suicide. Psychol Bull. (2011) 137:391–420. doi: 10.1037/a0022935 21443319

[79] Nicolaides NC Kyratzi E Lamprokostopoulou A Chrousos GP Charmandari E. Stress, the stress system and the role of glucocorticoids. Neuroimmunomodulation. (2015) 22:6–19. doi: 10.1159/000362736 25227402

[80] Lazarus RS. Coping theory and research: past, present, and future. Psychosom Med. (1993) 55:234–47. doi: 10.1097/00006842-199305000-00002 8346332

[81] Lazarus RS Folkman S. Transactional theory and research on emotions and coping. Eur J Pers. (1987) 1:141–69. doi: 10.1002/per.2410010304

[82] Cai L Choi K Hansen M Harrell L. Item response theory. Annu Rev Stat Its Appl. (2016) 3:297–321. doi: 10.1146/annurev-statistics-041715-033702

[83] Winsteps. Infit and outfit mean-square fit statistics(2020). Available online at: https://www.rasch.org/rmt/rmt82a.htm (Accessed November 30, 2024).

[84] Prinsen CAC Mokkink LB Bouter LM Alonso J Patrick DL de Vet HCW. COSMIN guideline for systematic reviews of patient-reported outcome measures. Qual Life Res. (2018) 27:1147–57. doi: 10.1007/s11136-018-1798-3 PMC5891568 29435801

[85] Polit DF. Getting serious about test–retest reliability: a critique of retest research and some recommendations. Qual Life Res. (2014) 23:1713–20. doi: 10.1007/s11136-014-0632-9 24504622

[86] Paiva CE Barroso EM Carneseca EC de Pádua Souza C dos Santos FT Mendoza López RV. A critical analysis of test-retest reliability in instrument validation studies of cancer patients under palliative care: a systematic review. BMC Med Res Methodol. (2014) 14:8. doi: 10.1186/1471-2288-14-8 24447633 PMC3899385

[87] Frost MH Reeve BB Liepa AM Stauffer JW Hays RD. What is sufficient evidence for the reliability and validity of patient-reported outcome measures? Value Heal. (2007) 10:S94–105. doi: 10.1111/j.1524-4733.2007.00272.x 17995479

[88] Grassi L Caruso R Sabato S Massarenti S Nanni MG. Psychosocial screening and assessment in oncology and palliative care settings. Front Psychol. (2015) 5:1485. doi: 10.3389/fpsyg.2014.01485 25709584 PMC4285729

[89] Organización Mundial de la Salud (OMS). Guía de bolsillo de la clasificación CIE-10. In: Clasificación De Los Trastornos Mentales y Del Comportamiento. Madrid: OMS (2008).

[90] Mitchell AJ Chan M Bhatti H Halton M Grassi L Johansen C. Prevalence of depression, anxiety, and adjustment disorder in oncological, haematological, and palliative-care settings: a meta-analysis of 94 interview-based studies. Lancet Oncol. (2011) 12:160–74. doi: 10.1016/S1470-2045(11)70002-X 21251875

[91] Grassi L Riba M. Introducing Multicultural Psycho-oncology. In: Grassi L Riba M, editors. Clinical Psycho-Oncology. John Wiley & Sons, Ltd (2012), 1–9. doi: 10.1002/9781119941101.ch1

[92] Surbone A. Bioethical challenges: understanding cultural differences andreducing health disparities. In: Grassi L Riba M, editors. Clinical Psycho-Oncology. John Wiley & Sons, Ltd (2012), 199–210. doi: 10.1002/9781119941101.ch15

[93] Choi BCK Pak AWP. A catalog of biases in questionnaires. Prev Chronic Dis. (2005) 2:A13. PMC1323316 15670466

[94] Sunderland M Batterham P Calear A Carragher N. Self-report scales for common mental disorders. In: Cambridge Handb. Clin. Assess. Diagnosis. Cambridge: Cambridge University Press (2019). p. 263–77. doi: 10.1017/9781108235433.019

[95] Arbus C Hergueta T Duburcq A Saleh A Le Guern M-E Robert P. Adjustment disorder with anxiety in old age: Comparing prevalence and clinical management in primary care and mental health care. Eur Psychiatry. (2014) 29:233–8. doi: 10.1016/j.eurpsy.2013.04.002 23769681

[96] Alvarado-Esquivel C Sifuentes-Alvarez A Salas-Martinez C. Adjustment disorder in pregnant women: prevalence and correlates in a northern Mexican City. J Clin Med Res. (2015) 7:775–80. doi: 10.14740/jocmr2275w PMC4554217 26346070

