# Supplementary material for: Development and validation of the “Adjustment Disorder Scale for Medically Ill Patients - ETAM”
Source: Front Psychiatry. 2025 Jan 30;16:1482888. doi: 10.3389/fpsyt.2025.1482888 (PMC11821628; doi:10.3389/fpsyt.2025.1482888)
Supplement: Supplementary file 1 [file Table1.docx]

Supplementary Material

# Supplementary Material 1: Final version of the Adjustment Disorder Scale for Medically Ill Patients (ETAM, for its acronym in Spanish). Items 11 and 20 were removed but are shown to maintain the numbering presented in the analyses of this manuscript.


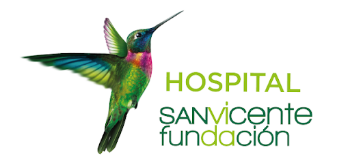

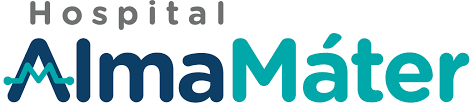

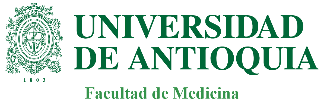


**ADJUSTMENT DISORDER SCALE FOR MEDICALLY ILL PATIENTS - ETAM**

**Name and Surname: ______________________________________________________________________________**

**Date: _____ /_____ /_____**

This questionnaire aims to identify how stressful situations are affecting you.

1. Please list ALL the situations that are currently stressing you:

|  |
| --- |
| (If nothing is stressing you at the moment, write the word "none" and you do not need to answer the rest of the questionnaire.) |

2. Now think about the stressful situations you just mentioned and what you have been feeling over the past 15 days. Based on that, mark with an X how often the following occurs:

|  | **Never** | **Rarely** | **Frequently** | **Always** |
| --- | --- | --- | --- | --- |
| 1. You have felt sad about these stressful situations |  |  |  |  |
| 2. Because of these stressful situations, you have felt like crying. |  |  |  |  |
| 3. Because of these stressful situations you have felt anxious. |  |  |  |  |
| 4. Due to these stressful situations you have been irritable or short-tempered. |  |  |  |  |
| 5. You have been afraid of what might happen in these stressful situations. |  |  |  |  |
| 6. You have been turning these stressful situations over and over in your mind. |  |  |  |  |
| 7. You have wished to die so as not to have to live these stressful situations. |  |  |  |  |
| 8. Because of these stressful situations you have thought of committing suicide. |  |  |  |  |
| 9. Because of these stressful situations, you have thought that you no longer have reasons to live. |  |  |  |  |
| 10. Because of these stressful situations, you have had trouble sleeping. |  |  |  |  |
| ~~11. You have felt guilty because of these stressful situations.~~ |  |  |  |  |

3. Continue thinking about the stressful situations you mentioned earlier and what you have been feeling over the past 15 days. Based on that, mark with an X how much you agree with the following statements:

|  | **STRONGLY disagree** | **Disagree** | **Agree** | **STRONGLY Agree** |
| --- | --- | --- | --- | --- |
| 12. Because of these stressful situations, your appetite has changed. |  |  |  |  |
| 13. Because of these stressful situations, you consume more alcohol, cigarettes or drugs than before. |  |  |  |  |
| 14. Because of these stressful situations you are less interested in following the treatments for your disease. |  |  |  |  |
| 15. It is difficult to control your emotional reactions to these stressful situations. |  |  |  |  |
| 16. The cause of your emotional discomfort is these stressful situations. |  |  |  |  |
| 17. Your emotional reactions to these stressful situations are more intense than what is normal for you. |  |  |  |  |
| 18. You feel that you have been defeated by these stressful situations. |  |  |  |  |
| 19. You feel that you need psychological help to cope with these stressful situations |  |  |  |  |
| ~~20. You believe that you can do something to handle these stressful situations~~ |  |  |  |  |
| 21. Because of these stressful situations, you have had problems in your relationship with other people |  |  |  |  |
| 22. Because of these stressful situations, you are less interested in taking care of your health. |  |  |  |  |

**Thank you very much for your answers!**

**Copyright © by University of Antioquia, Hospital Alma Mater de Antioquia and Fundación Hospitalaria San Vicente**
